# Joint wavelet decomposition of predictors and target variables for drought forecasting

**DOI:** 10.1038/s41598-026-58430-0

**Published:** 2026-06-20

**Authors:** Eliana Vivas, Chengyan Ji, Lelys Bravo de Guenni

**Affiliations:** 1 Faculty of Engineering and Sciences, Universidad Adolfo Ibáñez, Peñalolén, 7941169 Santiago, Chile; 2 Department of Statistics, University of Illinois at Urbana-Champaign, 703 S Wright St, Champaign, IL 61820 USA; 3 Department of Statistics, George Mason University, 4400 University Drive, Fairfax, VA 22030 USA

**Keywords:** Wavelet, Machine learning, Drought, Time series, Climate sciences, Hydrology, Mathematics and computing

## Abstract

We propose a leakage-free wavelet-based methodology for drought prediction that enables both competitive forecasting performance and improved interpretability of predictor–target relationships. Unlike standard time-series decomposition approaches, the wavelet transform is applied within moving windows of the past values, preventing the use of future information during model training. The method merges local and large-scale climate predictors across multiple temporal scales and is evaluated using 76 years of drought data from Illinois, USA. Predictive performance is assessed using RMSE, SMAPE, MASE, R^2^, and directional accuracy (DA), including uncertainty calculated with seasonal bootstrap confidence intervals. The proposed model frequently outperforms naïve and univariate wavelet baselines, achieving improvements of up to 26.5% in RMSE and 4.95% in SMAPE and higher directional accuracy in diverse scenarios compared to several benchmark methods. Through decomposing both predictors and target variables, the methodology helps characterize how different frequency components are associated with drought evolution, providing a more interpretable representation of hydroclimatic interactions and additional insights into the scale-dependent dynamics of drought processes.

## Introduction

A signal can be understood as a quantitative representation of a physical phenomenon that varies over time, space, or with respect to another variable and can take continuous or discrete values^1^. In the real world, this representation is usually a complex composition of multiple patterns, trends, and transient events occurring at different scales, whose important information can be located in either the time or frequency domain.

This is precisely where the wavelet transform becomes relevant. The wavelet transform allows the analysis of signals and images through multiresolution decomposition, capturing the frequency components and preserving their temporal location^2^.

This capability has become valuable in hydroclimatic prediction, an area in which researchers are increasingly employing wavelet-based methods to improve the prediction of phenomena associated with drought, such as those described in^3–11^, or phenomena associated with precipitation, such as those presented in^12–14^, and even renewable energy^15,16^.

The potential of wavelets to analyze complex dynamics is particularly useful in the study of drought. This phenomenon, considered a slow-evolving natural disaster whose ecological and economic impacts are becoming increasingly severe due to climate change^17,18^, is often represented by indices such as the Standardized Precipitation Index (SPI) and the Standardized Precipitation-Evapotranspiration Index (SPEI). By integrating precipitation and temperature, SPEI generates a signal that reflects the water balance and which in turn contains patterns and trends at multiple frequencies that traditional techniques do not usually separate, making decomposition methods such as wavelets relevant.

For instance,^7^ applies wavelet decomposition as a data preprocessing technique, transforming lagged SPI values into sub-signals at different frequency levels to enhance forecasting performance. Similarly,^5^ proposed a hybrid approach combining wavelet transform with machine learning models (Gaussian Process Regression, Cascade Neural Network, and Multi-Layer Perceptron) to predict the SPEI index. Taking this integration a step further,^19^ proposes a deep learning architecture, MultiWave, which embeds multi-resolution wavelet decomposition directly into the processing of multivariate time series, allowing specialized sub-models to handle distinct frequency bands. Similarly, the study by^20^ proposes hybrid models that combine deep learning with wavelet decomposition to forecast the Streamflow Drought Index (SDI) at 3-, 6-, and 12-month horizons. This approach enables the characterization of hydrological drought dynamics across short-, medium-, and long-term scales, supporting more effective water resource management.

Beyond forecasting, these multiscale techniques are also used to uncover complex physical relationships. For example,^6^ investigated the time- and scale-dependent relationship between meteorological (SPI) and hydrological (SRI) droughts, finding that their associations are dynamic and influenced by local factors.

A persistent recommendation emerges from these studies: the need to enrich models with more diverse data inputs. Researchers consistently suggest incorporating additional drought indices, large-scale climatic signals (such as NAO and NINO), and other meteorological variables as a potential strategy to improve model robustness and generalizability^5–7^. This consensus highlights a clear avenue for advancing drought prediction; however, effectively leveraging this richer information requires methodological frameworks capable of representing complex, scale-dependent relationships among predictors and targets.

In this regard, the scientific literature increasingly supports the integration of signal decomposition techniques with machine learning models. However, recent studies have highlighted the potential for data leakage in hybrid time series forecasting approaches that employ discrete wavelet transform. Importantly, these studies also describe some approaches to prevent data leakage while preserving model robustness^21,22^.

Motivated by this challenge and by methodological gaps in existing wavelet-based drought forecasting studies, this work aims to develop and evaluate a unified multiscale forecasting methodology that systematically incorporates wavelet-based decomposition into both drought indices and climate predictors. Although hybrid wavelet–machine learning frameworks have shown promising results, most existing approaches decompose either the target drought index or the predictor variables, rarely both simultaneously, which may limit the representation of scale-dependent interactions.

The proposed methodology is intended to capture relationships specific to distinct temporal scales, improve model robustness and interpretability, and identify the dominant frequency components through which climate predictors associated with drought dynamics. Furthermore, a moving-window strategy is employed to mitigate information leakage and ensure strictly causal forecasting conditions.

The main novelty of this study lies in the simultaneous and causal wavelet decomposition of both drought indices and predictor variables, facilitating scale-consistent learning of hydroclimatic interactions.

The methodology is validated using SPI and SPEI indices and benchmarked against traditional and hybrid forecasting approaches to assess its effectiveness in capturing complex multiscale drought processes.

The remainder of this paper is organized as follows. Section 2 presents a brief review of the relevant literature. Section 3 describes the data and methodology employed. Section 4 discusses the performance of the proposed methodology. Section 5 provides a comparative analysis against several widely used benchmark forecasting models. Finally, Section 6 summarizes the main conclusions and outlines potential directions for future research.

## Literature review

The literature review was conducted with a primary focus on drought forecasting studies, particularly those that integrate wavelet-based decomposition techniques with statistical and machine learning models. In addition to drought-specific applications, selected contributions from related forecasting domains were also considered when they provided relevant methodological insights. In this context, the study by^21^–although focused on wind speed forecasting–and the study of^22^ about water demand forecasting were included due to its in-depth analysis of data leakage issues associated with wavelet-based decomposition and sampling strategies, which are directly applicable to time series forecasting problems. Furthermore, the methodological treatment and benchmarking role of naïve models, as discussed by^3^, were taken into account to ensure robust and transparent evaluation practices. Together, these studies informed the design of the data preprocessing, modeling, and evaluation framework adopted in this work. While the broader bibliographic review encompasses a wider range of studies discussed throughout the paper, this section highlights twelve representative contributions summarized in Table 1, which collectively capture the key methodological trends and challenges relevant to wavelet-based forecasting of drought indices.

**Table 1:** Summary of representative studies on wavelet-based and machine learning approaches for drought forecasting.

Reference	Primary method	Data resolution	Target index	Forecast horizon
^3^	DWT + NN	Monthly	PDSI	1–12 months
^22^	WDDFF	Daily	Water demand	1-14 days
^23^	DWT / ARIMA / ANN	Monthly	SPI, SIAP	1 month
^4^	WD / ML	Monthly	sc-PDSI	1–6 months
^5^	DWT / GPR / NN	Monthly	SPEI (3,12,24)	1–6 months
^11^	TQWT / MODWT / ML	Monthly	SPEI (12, 18)	6–24 months
^6^	ICEEMDAN / TDIC	Monthly	SPI / SRI	Inter-annual
^24^	MLR	Monthly	SPEI / SPI	1-12 months
^7^	WD / SVM / LSTM	Monthly	SPI3, SPI12	Seasonal
^8^	DWT / SVM	Monthly	SPI (3–12)	1–12 months
^9^	VMD / GWO / SVM	Monthly	SGI	3-12 months
^21^	MODWT	Hourly	Wind speed	3–12 hours
^20^	QRNN	Monthly	SDI (3,6,12)	1-12 months
^25^	WD / LSTM	Monthly	SPI (3-12)	1 month
^26^	SVR/ SARIMA	Monthly	SPEI (1-24)	1 month

## Data and methodology

### Study area, drought indices, and climate predictors

This study examines drought conditions in Illinois using monthly aggregated data from January 1950 to April 2025. The target variables are the Standardized Precipitation Index (SPI) and the Standardized Precipitation-Evapotranspiration Index (SPEI), both obtained from the^27^. These indices capture precipitation anomalies and the influence of evaporative demand, respectively.

To enhance model performance, a solar activity predictor–specifically the 10.7 cm Solar Flux–was also included, motivated by recent findings that suggest an inverse relationship between solar radiation and drought indices, particularly SPEI, due to its sensitivity to temperature-driven evaporation^24,28,29^.

Additional large-scale climate predictors with known teleconnections to precipitation and temperature in North America–such as ONI, NAO, PDO, PNA, WP, and Niño 3.4–were sourced from^30^ and other public datasets,^27^. All time series were validated to ensure completeness and showed no missing values throughout the study period. Table 2 summarizes the variables used, their roles, brief descriptions, and data sources.

**Table 2:** Summary of target drought indices and associated climate predictors used for model development.

Type	Index	Description	Source
Target	SPI	Standardized index of precipitation anomalies across multiple accumulation periods.	^27^
	SPEI	Standardized drought index incorporating precipitation and temperature effects.	
Macroclimatic covariates	ONI	Index based on sea surface temperature anomalies in the Niño 3.4 region.	^31^
	NAO	Atmospheric circulation index over the North Atlantic region.	^32^
	PDO	Long-term Pacific climate variability index derived from sea surface temperature anomalies.	^33^
	Niño 3.4	Sea surface temperature anomaly index in the central equatorial Pacific.	^34^
	WP	Teleconnection index describing pressure anomalies over the North Pacific.	^35^
	PNA	Teleconnection index representing atmospheric variability over North America.	^36^
	Solar Flux	F10.7 solar activity index based on radio emissions.	^37^
Local covariates	Precipitation	Total monthly precipitation expressed as liquid water equivalent.	^27^
	Temperature	Mean monthly near-surface air temperature.	

**Figure 1:**
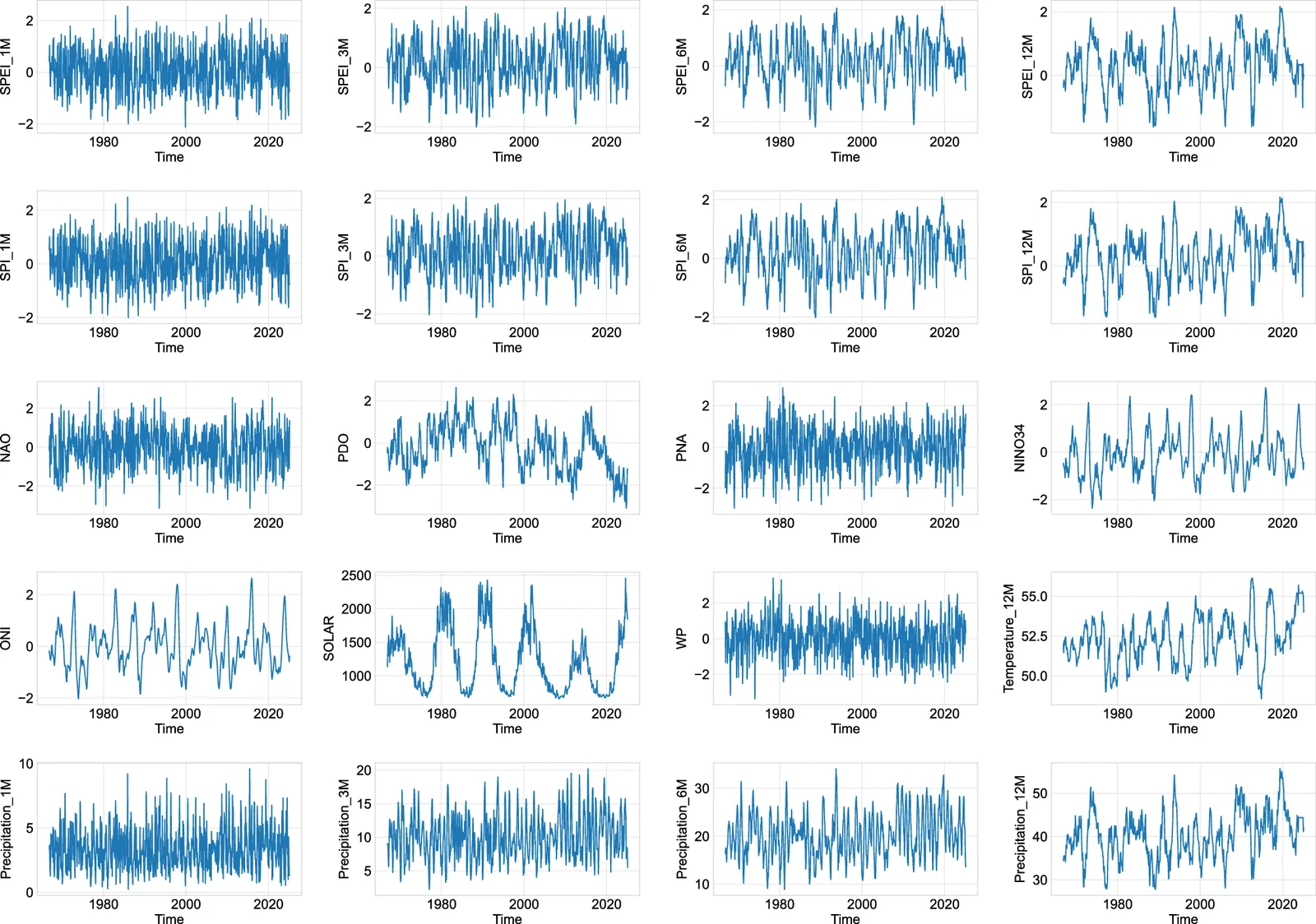
Temporal dynamics of drought indices and associated climate predictors in Illinois over the 1950–2025 period.

All drought indices and local climate variables presented in Table 2 were obtained at multiple accumulation periods (1, 3, 6, and 12 months). Variables are denoted by the suffixes _1M, _3M, _6M, and _12M, which indicate the corresponding accumulation or averaging windows. Fig. 1 shows the time series of the drought indices (SPEI and SPI) and their predictors for the 1950–2025 period.

Between the **first and third rows**, the volatile nature and the lack of clearly persistent trends in the drought indices and the NAO are observed. The **third row** also illustrates the behavior of the PDO with its long-term phases; the high variability of the PNA fluctuating around the mean; and the pronounced ENSO cycles of the Niño 3.4 index, where El Niño (positive) and La Niña (negative) events are visible. In the **fourth row**, the ONI index reflects the same ENSO dynamics as Niño 3.4 but with smoother transitions, as it is a moving average. Next to it, SOLAR (solar flux) exhibits a clear cyclical pattern of approximately 11 years, corresponding to solar activity, and the WP index displays high-frequency variability with abrupt changes, characteristic of atmospheric teleconnections. Finally, the **fifth row** illustrates the behavior of precipitation, which is highly variable.

**Figure 2:**
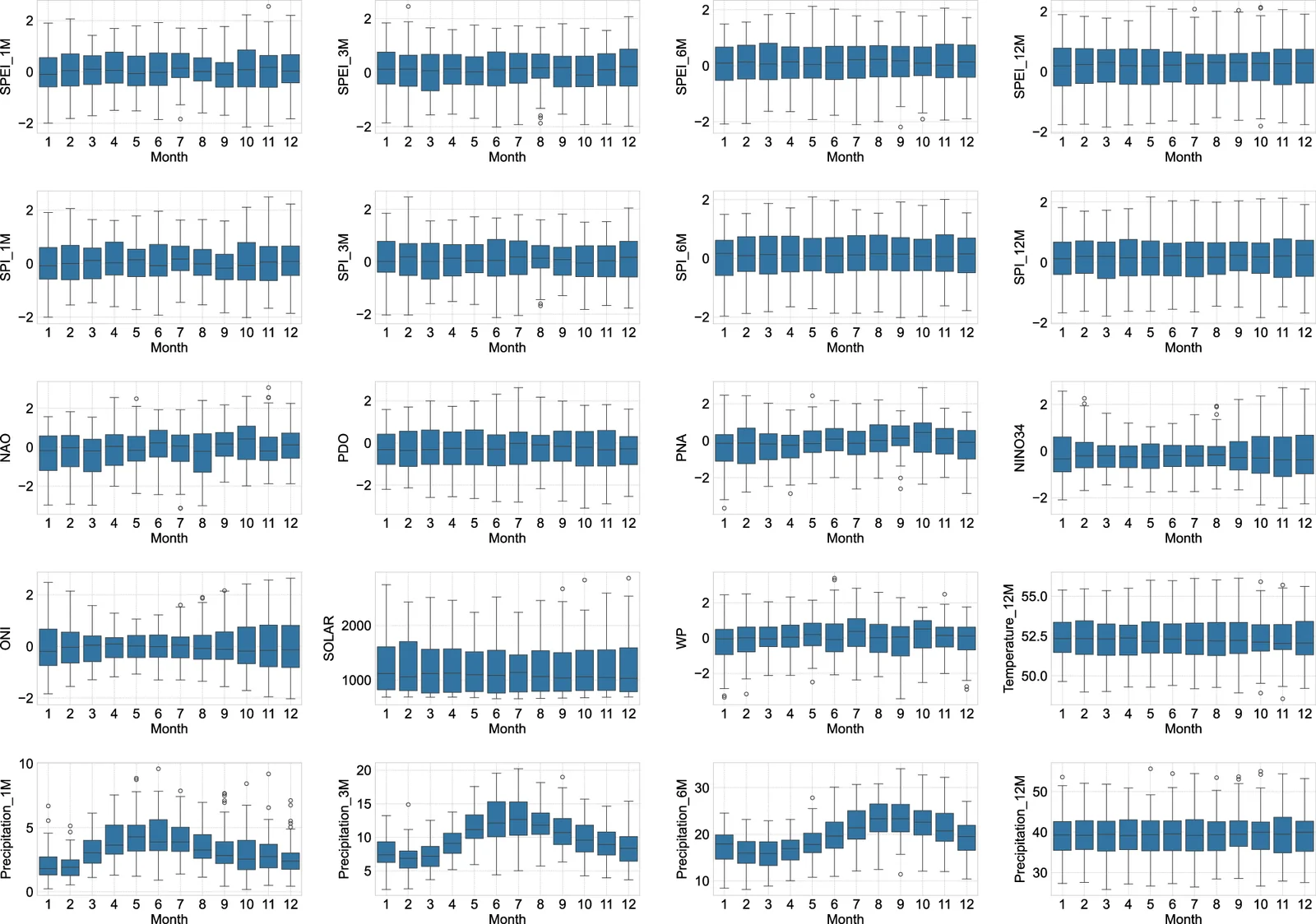
Monthly boxplots of drought indices and climate predictor variables for the 1950–2025 period, illustrating seasonal variability, dispersion, and the presence of extreme values across months.

Complementing the description of the time series, Fig. 2 presents monthly boxplots for each variable, allowing for an analysis of their seasonal distribution from January to December. These plots make it possible to identify changes in the median, dispersion, the presence of outliers, and potential seasonal asymmetries. The figure shows that while the drought indices (SPI and SPEI) do not exhibit a marked seasonality, several climatic predictors do show distinct monthly patterns. Specifically, NAO and PNA display a slight seasonality, whereas ONI and Niño 3.4 show minor increases in their median and dispersion between September and January. Furthermore, the WP index exhibits the highest number of outliers, particularly at the extremes of the monthly series. These findings suggest that the climatic influence on drought may have an indirect seasonal component, mediated by the monthly variations of the considered predictors.

### Proposed multiscale forecasting methodology

**Figure 3:**
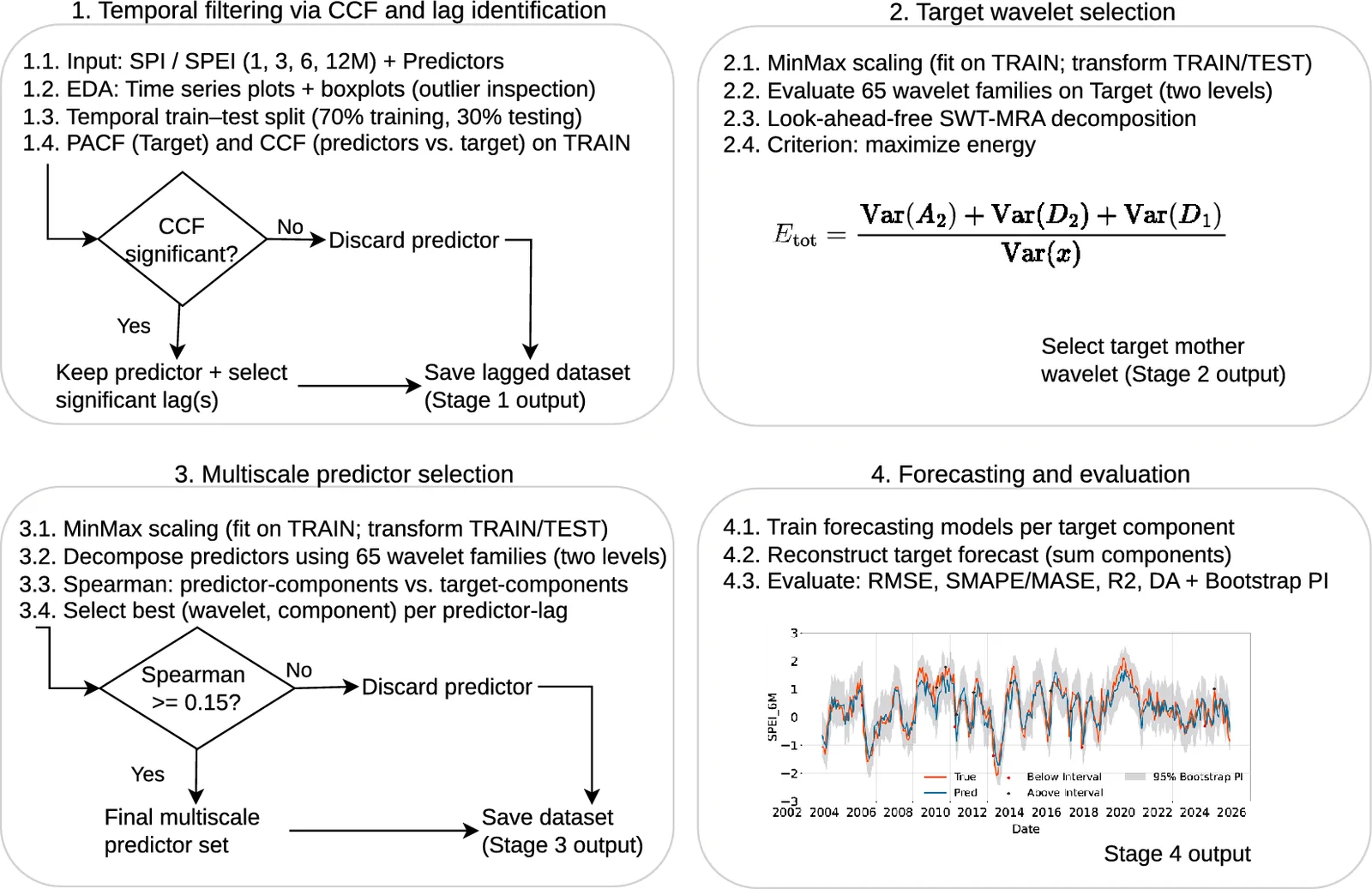
Integrated multiscale methodology for systematic predictor selection and drought forecasting, applicable across all targets and accumulation scales.

As shown in Fig. 3, the methodology is organized into four sequential stages: (1) temporal filtering and lag identification via CCF; (2) selection of the optimal mother wavelet for the target based on energy preservation; (3) identification of scale-specific predictor-target pairs through an exhaustive Spearman correlation analysis; and (4) forecasting using the selected multiscale feature set. The procedure is illustrated in step 4 for a representative target and accumulation period within the figure, and the same approach is applied across all drought indices and accumulation scales considered in this study. Furthermore, a 70% training and 30% testing partition was consistently employed for all methodology stages.

### Temporal filtering via cross-correlation and lag identification

The significance of the autocorrelation (ACF), partial autocorrelation (PACF), and cross-correlation (CCF) analyses was assessed using 95% confidence intervals, computed based on the effective sample size. To ensure that correlation estimates reflected genuine temporal relationships rather than shared trends^38^, differencing was applied selectively depending on the accumulation scale of the drought indices.

At the 1-month scale, differencing was required to mitigate the dominance of short-term persistence and trend effects in the raw SPI and SPEI series, thereby highlighting month-to-month fluctuations that drive immediate drought responses. Under these conditions, ACF, PACF, and CCF analyses on the differenced series facilitated the identification of relevant lagged relationships.

In contrast, for the 3-, 6-, and 12-month accumulation periods, SPI and SPEI inherently embed temporal integration and long-term memory. At these scales, the original (non-differenced) series were retained, as differencing tended to suppress the accumulated signal characteristic of persistent drought processes. The ACF and PACF analyses of these series (Table 3) revealed strong persistence, evidenced by the slow decay of correlation coefficients across lags.

**Table 3:** Summary of selected lags for drought indices across different accumulation horizons. The symbol ”-” indicates that no significant lag was identified for the corresponding predictor–target combination.

Type	Predictor	1M	3M	6M	12M
**Panel A: SPEI**					
Macroclimatics	NAO	9, 13, 19	6	–	7, 12, 14, 18
	NIÑO34	20	1	14	4, 11, 15
	ONI	–	1, 2	8, 9	3, 4, 5, 14, 16
	PDO	7, 16	8, 14, 17	17	–
	PNA	4, 5, 11, 12	6, 20	3, 5, 6	5
	SOLAR	1, 2, 7, 8, 12	2, 5, 16	2, 7, 8, 13	8, 10, 19
	WP	2, 5, 11, 12, 14	2, 3, 12	2, 10, 12, 15	6, 8
Locals	Precipitation_1M	1, 5, 6	1, 2	1, 2	1, 2, 19
	Precipitation_3M	1, 2, 3, 4	2, 3	2	3, 18
	Precipitation_6M	5, 6	3, 6	1, 6, 12	6, 17
	Precipitation_12M	11, 12, 13	9, 12, 20	6, 12	1, 5, 12, 15
	Temperature_1M	–	–	–	–
	Temperature_3M	–	–	–	–
	Temperature_6M	–	–	–	–
	Temperature_12M	5	10, 13, 18	6, 7	1, 6, 7, 13
Auto regressive	SPEI_1M	1–7	–	–	–
	SPEI_3M	–	1–7, 10	–	–
	SPEI_6M	–	–	1, 2, 6, 12, 18	–
	SPEI_12M	–	–	–	12, 24
**Panel B: SPI**					
Macroclimatics	NAO	9, 19	6, 10	9	7, 12
	NIÑO34	20	1	14	–
	ONI	–	11	8, 9	3, 4, 5, 14
	PDO	16, 17	8, 14, 17	–	5
	PNA	4, 5, 11, 12	6, 12, 20	1, 3, 5, 6	5
	SOLAR	1, 2, 7, 8, 12	2, 5, 16	2, 7, 8, 13	8, 13, 19
	WP	5, 11, 14, 15, 17	2, 12	11, 12, 15	5, 6, 7, 12
Locals	Precipitation_1M	1, 6	1	1, 2	1, 2, 6, 19
	Precipitation_3M	1, 2, 3	2, 3	3	3, 18
	Precipitation_6M	5, 6	3, 6	1, 6, 12	6, 17, 18
	Precipitation_12M	11, 12, 13	9, 12, 20	2, 12	1, 5, 12, 13, 15
	Temperature_1M	–	–	–	–
	Temperature_3M	–	–	–	–
	Temperature_6M	–	–	–	–
	Temperature_12M	12	10, 13, 18	7, 12	1, 6, 13, 18
Auto regressive	SPI_1M	1–10	–	–	–
	SPI_3M	–	1–7, 10	–	–
	SPI_6M	–	–	1, 2, 6, 12, 18	–
	SPI_12M	–	–	–	12, 24

Cross-correlation analyses were then conducted between each predictor and the corresponding target index, and lags whose correlation coefficients exceeded the 95% confidence bounds, approximated as $\pm 1.96/\sqrt{n}$, were retained as candidate predictors for the subsequent modeling stage.

### Wavelet decomposition strategy

To preserve the causal structure of the time series and prevent temporal data leakage^21,22^, we implemented a look-ahead-free variant of the Stationary Wavelet Transform (SWT), hereafter referred to as a causal SWT. In contrast to standard SWT decompositions computed on the full signal, our implementation computes the decomposition at each time index t using only observations available up to and including t, thereby avoiding look-ahead bias in forecasting settings^39,40^. Specifically, SWT is applied over rolling causal windows (expanding only at the beginning of the record), and the window length is adjusted to satisfy the SWT length constraint (i.e., a multiple of $2^L$ for decomposition level *L*) using left-side padding only. When padding is required, the procedure first uses available historical observations and, if insufficient, repeats the earliest value in the window (causal constant padding). The SWT components are reconstructed via inverse SWT by isolating the approximation $A_L$ and each detail $D_j$; the value retained at time t corresponds to the last sample of the reconstructed window. Since SWT admits perfect reconstruction for each padded window, the sum $A_L(t)+\sum _{j=1}^{L} D_j(t)$ matches the original observation *x*(*t*) up to numerical precision, ensuring no information loss at the retained time index.

### Methodology for optimal target wavelet selection

The optimal mother wavelet for each target variable was selected through a variance-preservation criterion. While the SWT theoretical framework allows for perfect reconstruction, the practical implementation of a causal SWT–which relies on sliding windows and boundary padding–can introduce subtle scale-specific distortions. To minimize these effects, we evaluated 65 candidate wavelet families and computed the energy preservation ratio as: $E_{total} = \frac{Var(A_2) + Var(D_2) + Var(D_1)}{Var(Y_{original})}$ where $A_2$ represents the reconstructed approximation and $D_2, D_1$ the reconstructed details at each time step *t*. The wavelet yielding the highest $E_{total}$ was selected, ensuring that the decomposed components collectively retain the maximum possible variance of the original signal under the causal constraint. This criterion helps preserve a multiscale representation that remains as faithful as possible to the original drought dynamics, following energy-based selection logic reported in recent literature^41^.

**Table 4:** Optimal mother wavelet families identified for the target drought indices at different temporal accumulation periods.

Index	1M	3M	6M	12M
SPEI	db8	db8	db8	sym6
SPI	db8	db8	db8	sym6

Table 4 summarizes the optimal mother wavelet families selected for the target drought indices at different accumulation periods using the energy-based criterion. The selection was performed by evaluating a set of wavelet families, including Daubechies, Symlets, Coiflets, and Biorthogonal wavelets.

The energy-based selection criterion consistently identified db8 (Daubechies wavelet with eight vanishing moments) as the optimal mother wavelet for shorter accumulation periods (1M, 3M, and 6M). The number of vanishing moments characterizes a wavelet’s insensitivity to smooth polynomial trends: a wavelet with *N* vanishing moments yields zero response to polynomials up to degree *N-1*. Consequently, wavelets with higher vanishing moments, such as db8, reduce the influence of smooth low-order components in the detail coefficients, allowing the decomposition to emphasize transient and multiscale fluctuations that dominate short-term drought variability. Under the proposed look-ahead-free SWT framework, this property was associated with improved preservation of the variance structure across approximation and detail components. Moreover, the compact support and relatively high smoothness of db8 enable an effective representation of both localized anomalies and broader oscillatory patterns characteristic of short- to medium-term drought dynamics.

At the 12-month scale, the selection shifts to sym6, reflecting the smoother, low-frequency nature of long-term drought dynamics. The increased symmetry and near-linear phase response of Symlet wavelets help minimize phase distortions that can disproportionately affect the reconstruction of highly aggregated signals, making sym6 particularly suitable for representing gradual, trend-like drought evolution.

Notably, the optimal wavelet was identical for both SPEI and SPI at each accumulation period, suggesting that the temporal aggregation scale may play a dominant role in shaping the spectral characteristics relevant for wavelet decomposition. This consistency provides additional support for the robustness of the proposed energy-based wavelet selection framework.

### Methodology for optimal predictor wavelet selection

Following the energy-based selection of the optimal wavelet for the target series, a correlation-maximization strategy was applied to select predictor-specific wavelet families. In contrast to conventional approaches that rely on a single wavelet family for all variables^40,42,43^, the proposed method accounts for the fact that different climate predictors may exhibit distinct spectral characteristics and, consequently, different optimal representations in the frequency domain.

For each predictor $X_i$, the time series was decomposed using 65 candidate wavelet families, and the Spearman rank correlation coefficient was computed between its decomposed components and the corresponding components of the target series, previously decomposed using its optimal wavelet. The wavelet family yielding the strongest overall predictor–target association–quantified as the maximum Spearman correlation across all corresponding decomposed components–was selected for each predictor.

This predictor-specific strategy is conceptually related to wavelet coherence analysis^44,45^, as it aims to strengthen scale-dependent associations between predictor and target components. However, rather than quantifying coherence within a fixed wavelet domain, the proposed approach explicitly optimizes the wavelet decomposition basis itself to enhance predictor–target correspondence.

Following wavelet decomposition, an initial feature selection was performed based on Spearman correlation thresholds between predictor and target components. Predictors whose decomposed components exhibited absolute correlations below $|\rho _s| \ge 0.10$ for short-term targets (1M) or below $|\rho _s| \ge 0.15$ for longer accumulation periods (3M, 6M, and 12M) were excluded from subsequent modeling stages. While these thresholds are below the conventional “medium effect” benchmark ($|\rho _s| \approx 0.30$) proposed by Cohen^46^, this relatively inclusive criterion was deliberately adopted to mitigate the risks associated with aggressive univariate filtering. As noted by Guyon and Elisseeff^47^, stringent correlation-based selection may prematurely discard variables that exhibit weak individual associations with the target but contribute meaningfully to predictive performance when combined with other features in a multivariate context. By establishing a lower threshold, the feature space retains potentially informative predictors whose relevance can be assessed more comprehensively during the modeling phase, where interactions and nonlinear relationships are explicitly captured.

**Figure 4:**
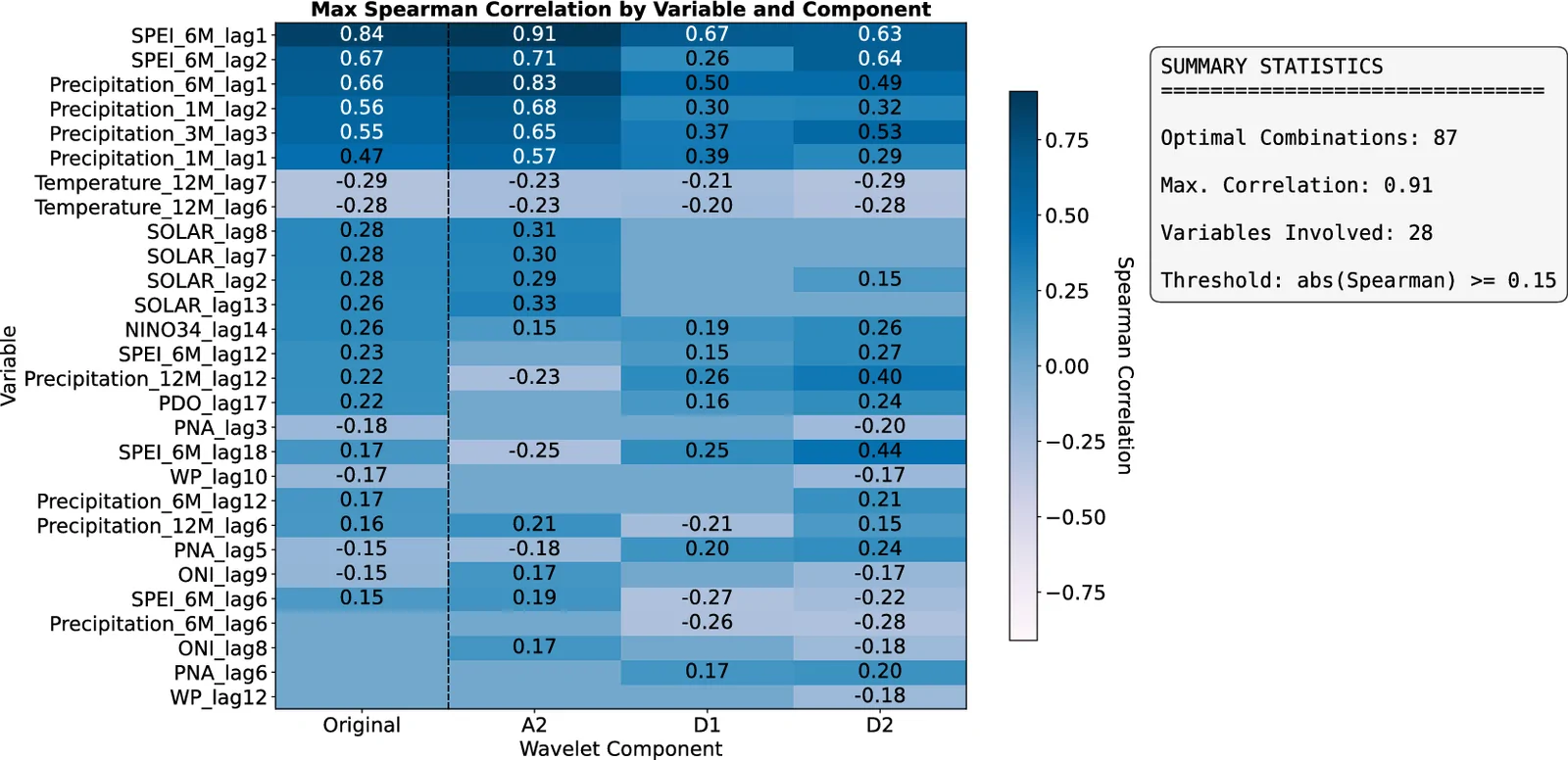
Heatmap of statistically significant maximum Spearman correlations between lagged hydroclimatic predictors and the original/SWT-decomposed SPEI-6M series. The analysis highlights scale-specific predictor–target associations across the A2, D1, and D2 components.

**Figure 5:**
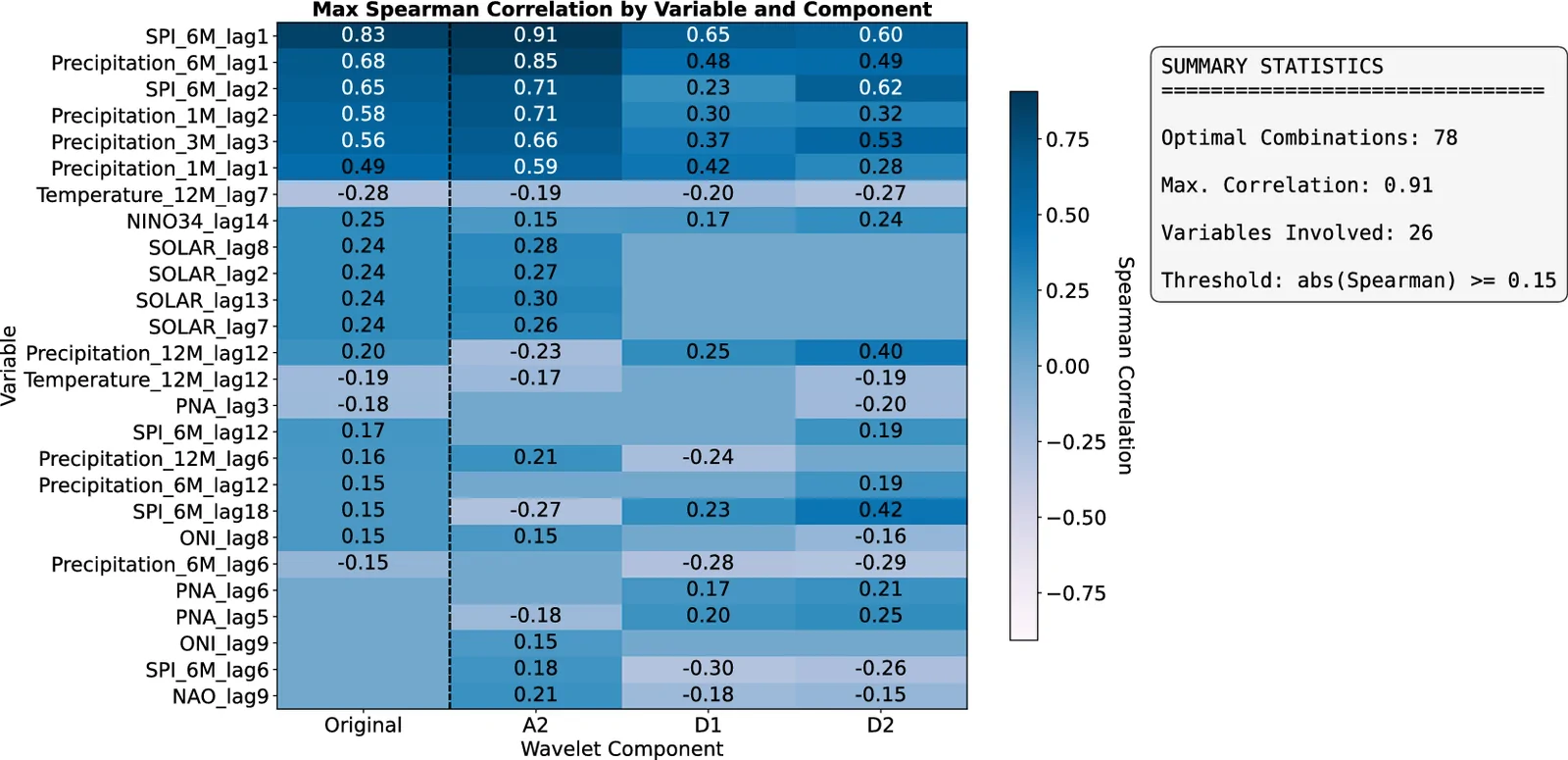
Heatmap of statistically significant maximum Spearman correlations between lagged hydro-climatic predictors and the original and SWT-decomposed SPI-6M series (A2, D1, and D2). The analysis highlights scale-specific predictor–target associations across the A2, D1, and D2 components.

The wavelet-based correlation analysis results are presented in Fig. 4 and 5, which illustrate the maximum Spearman rank correlation coefficients obtained between the decomposed components of each climate predictor and the corresponding components of the target drought index. Results are displayed for SPEI_6M and SPI_6M as representative cases; analogous patterns were consistently observed across all accumulation periods. The analysis reveals several noteworthy findings concerning the temporal scale-dependent associations between predictors and drought indices.

*Component distribution*: Both SPEI and SPI show strong representation across all wavelet components, with the approximation component (A2) demonstrating the highest Spearman correlation values, indicating its importance in capturing the long-term trends in drought dynamics.

*Autoregressive dominance*: The highest Spearman correlation values are consistently observed in the first lag of the target variables, confirming the strong autoregressive nature of drought indices and their importance in predictive modeling.

*Macroclimatic teleconnections*: External predictors (NAO, PNA, ONI, SOLAR, NIñO34, WP) show moderate but consistent Spearman correlation values across different lag structures, validating their utility in drought forecasting applications.

Table 5 summarizes the distribution of the top-selected wavelet families across drought indices and accumulation periods. For each index–period combination, the column “Total predictors” indicates the total number of significant covariates selected across all wavelet components of the target variable, while the remaining columns report the three most frequently selected wavelet families, including their counts and relative percentages.

For example, in the SPEI 1M case, a total of 71 predictors were selected across all decomposition levels, of which 46 (64.8%) correspond to Daubechies wavelets, 16 (22.5%) to biorthogonal wavelets, and 7 (9.9%) to Coiflets.

Overall, Daubechies wavelets dominate across most configurations, indicating their strong suitability for representing drought-related signals, particularly at shorter accumulation periods.

**Table 5:** Summary of the most frequently selected wavelet families across drought indices and accumulation periods.

Index	Period	Total predictors	Top wavelet families		
			1st	2nd	3rd
SPEI	1M	71	db (46, 64.8%)	bior (16, 22.5%)	coif (7, 9.9%)
	3M	49	db (43, 87.8%)	coif (5, 10.2%)	sym (1, 2.0%)
	6M	62	db (30, 47.6%)	bior (29, 46.0%)	rbio (2, 3.2%)
	12M	96	db (45, 46.9%)	bior (44, 45.8%)	coif (7, 7.3%)
SPI	1M	59	db (35, 59.3%)	bior (17, 28.8%)	coif (7, 11.9%)
	3M	44	db (39, 88.6%)	coif (4, 9.1%)	sym (1, 2.3%)
	6M	57	db (27, 47.4%)	bior (24, 42.1%)	coif (2, 3.5%)
	12M	83	db (39, 47.0%)	bior (36, 43.4%)	coif (4, 4.8%)

Values in parentheses indicate (count, percentage). Wavelet families were selected for Causal SWT decomposition of climate predictors used in drought index forecasting models across different temporal accumulation scales

### Evaluation metrics

To evaluate the accuracy of the drought forecasts, we rely on well-established statistical indicators. In particular, RMSE, SMAPE, MASE, DA, and $R^{2}$, are employed, as they provide complementary perspectives on model performance. Table 6 provides a detailed summary of their definitions.

**Table 6:** Summary and mathematical formulation of the evaluation metrics used for drought forecasting performance assessment.

Metric	Description	Equation
RMSE	Penalizes larger errors more strongly by squaring them before averaging. Useful for capturing the overall prediction accuracy^48^.	$\textrm{RMSE}=\sqrt{\frac{1}{N}\sum _{t=1}^{N}(y_t-\hat{y}_t)^2}$
SMAPE	Symmetrical Mean Absolute Percentage Error.	$\textrm{sMAPE}=\frac{100}{N}\sum _{t=1}^{N}\frac{2|\hat{y}_t-y_t|}{|y_t|+|\hat{y}_t|+\varepsilon }$
MASE	The Mean Absolute Scaled Error is computed using a naïve persistence benchmark (*m=1*), where forecasts are compared against the previous observation^48^.	$\textrm{MASE}=\frac{\frac{1}{N}\sum _{t=1}^{N}|y_t-\hat{y}_t|}{\frac{1}{T-m}\sum _{t=m+1}^{T}|y^{\textrm{train}}_t-y^{\textrm{train}}_{t-m}|}$
DA	Directional Accuracy. Evaluates whether a model correctly captures the direction of change of a time series^49,50^.	$\textrm{DA}=\frac{1}{N-1}\sum _{t=2}^{N}\mathbb {I}\big (\textrm{sign}(\Delta y_t)=\textrm{sign}(\Delta \hat{y}_t)\big )$
$R^{2}$	Indicates the proportion of variance in the observations explained by the model. Values closer to 1 indicate better fit.	$R^2=1-\frac{\sum _{t=1}^{N}(y_t-\hat{y}_t)^2}{\sum _{t=1}^{N}(y_t-\bar{y})^2}$

where $y_t$ denotes the observed value, $\hat{y}_t$ the predicted value, *N* the number of observations in the evaluation set, $\bar{y}$ the mean of observed values, $y^{\textrm{train}}_t$ the training series, *T* its length, *m* the lag of the naïve benchmark (here *m=1*), $\Delta y_t=y_t-y_{t-1}$ and $\Delta \hat{y}_t=\hat{y}_t-\hat{y}_{t-1}$, and *\varepsilon >0* a small constant to stabilize sMAPE near zero

### Benchmark analysis of forecasting models

A total of eight algorithms were evaluated. Two of them are based on the traditional DWT framework: one applying the decomposition exclusively to the target series (WaveDWT), and another performs decomposition on both the target and predictor variables (BiwaveDWT). Six additional algorithms were included as benchmark models.

The Naïve model is widely used as a baseline reference in drought forecasting literature. Ridge and ElasticNet represent regularized linear regressors (L2 and mixed L1–L2 penalties, respectively), commonly employed in hydroclimatic prediction problems involving correlated predictors. Support Vector Machines (SVR) provide a robust nonlinear alternative through linear, radial basis function (RBF), and polynomial kernels. HistGBM is a tree-based gradient boosting model capable of capturing nonlinear relationships and variable interactions without requiring extensive feature engineering.

Finally, WaveSWT applies the previously described causal stationary wavelet decomposition only to the target series, whereas the proposed approach (BiwaveSWT) simultaneously decomposes both the target and predictor variables, enabling scale-consistent feature extraction while mitigating cross-scale mixing effects.

Neural network models were also considered; however, during model development, they consistently showed strong overfitting under our data regime and rolling validation scheme. Consequently, they were excluded from the final benchmark to maintain robust, reproducible comparisons. Additional classical methods (e.g., Random Forest, Seasonal Naïve baseline, and PCR) were also tested, but their results were not as competitive as those reported here and thus are omitted for conciseness.

#### Model formulation

The main benchmark models considered in this study are briefly summarized below.

Ridge Regression Ridge regression minimizes the residual sum of squares with an L2 regularization term:

1 $$\begin{aligned} \min _{\beta } \left\| \textbf{y}-\textbf{X}\beta \right\| _2^2 + \alpha \left\| \beta \right\| _2^2 \end{aligned}$$

where $\textbf{X}\in \mathbb {R}^{n\times p}$ is the predictor matrix, $\textbf{y}\in \mathbb {R}^{n}$ is the target vector, *β* are the regression coefficients, and $\alpha \ge 0$ controls the strength of the L2 regularization.

Elastic Net Elastic Net combines L1 and L2 regularization penalties:

2 $$\begin{aligned} \min _{\beta } \frac{1}{2n} \left\| \textbf{y}-\textbf{X}\beta \right\| _2^2 + \alpha \lambda \left\| \beta \right\| _1 + \frac{\alpha (1-\lambda )}{2} \left\| \beta \right\| _2^2 \end{aligned}$$

where $\lambda \in [0,1]$ controls the balance between L1 and L2 penalties, and *α* defines the overall regularization strength.

Support Vector Regression (SVR) SVR minimizes the following objective function:

3 $$\begin{aligned} \min _{w,b} \frac{1}{2} \left\| w \right\| ^2 + C \sum _{i=1}^{n} (\xi _i+\xi _i^*) \end{aligned}$$

subject to

4 $$\begin{aligned} |y_i-(\langle w,\phi (x_i)\rangle +b)| \le \epsilon +\xi _i, \qquad \xi _i,\xi _i^* \ge 0 \end{aligned}$$

where *C* controls the penalty assigned to prediction errors, *ε* defines the insensitive loss region, $\xi _i$ and $\xi _i^*$ are slack variables, and $\phi (\cdot )$ maps the predictors into a higher-dimensional feature space through a kernel function $K(x_i,x_j)$.

Histogram-based Gradient Boosting (HistGBM) HistGBM constructs an additive ensemble model iteratively:

5 $$\begin{aligned} F_m(x) = F_{m-1}(x) + \eta \, h_m(x) \end{aligned}$$

where $F_m(x)$ is the model at iteration *m*, $h_m(x)$ are weak learners (decision trees) fitted to the gradients of the loss function, and *η* is the learning rate.

Naïve Forecasting Model The naïve forecasting model assumes that the future value is equal to the most recent observation:

6 $$\begin{aligned} \hat{y}_t = y_{t-1} \end{aligned}$$

where $\hat{y}_t$ is the forecast at time *t*, and $y_{t-1}$ is the previously observed value. This approach is commonly used as a persistence baseline in time series forecasting studies.

For all models, hyperparameters (Table 7) were selected via a consistent and leakage-free tuning procedure. Specifically, the time series is first sorted and split into training and test sets using a chronological holdout (test size = 30%). Model selection is then performed on the training set using grid search with time-series cross-validation (TimeSeriesSplit, three folds), optimizing negative mean squared error. Predictors are standardized for models that require scaling (e.g., linear models and SVR), and the target is also standardized during fitting and subsequently inverse-transformed so that all metrics are computed in the original units. In addition, the implementation stores both training and test predictions alongside their timestamps, enabling consistent downstream visualization and error analysis.

**Table 7:** Summary of machine learning models and hyperparameter configurations explored in this study.

**Model**	**Description**	**Hyperparameters (Selected ranges)**
Ridge	Linear regression with L2 regularization.	$\alpha \in [10^{-3}, 10^{3}]$ (log-scale)
Elastic Net	Linear regression combining L1 and L2 regularization.	$\alpha \in [10^{-3}, 10^{1}]$ $\lambda \in \{0.2, 0.5, 0.8\}$
SVM (SVR)	Support Vector Regression.	$C \in \{0.1, 1, 5, 10\}$ $\epsilon \in \{0.05, 0.1, 0.2, 0.3\}$ kernel $\in \{$linear, rbf*\}* $\gamma \in \{$scale, 0.001, 0.01*\}*
HistGBM	Gradient boosting trees.	learning_rate (*η*) $\in \{0.03, 0.1\}$ max_leaf_nodes $\in \{15, 31\}$ max_depth = default
Naïve	Persistence baseline.	No hyperparameters

## Performance analysis

**Figure 6:**
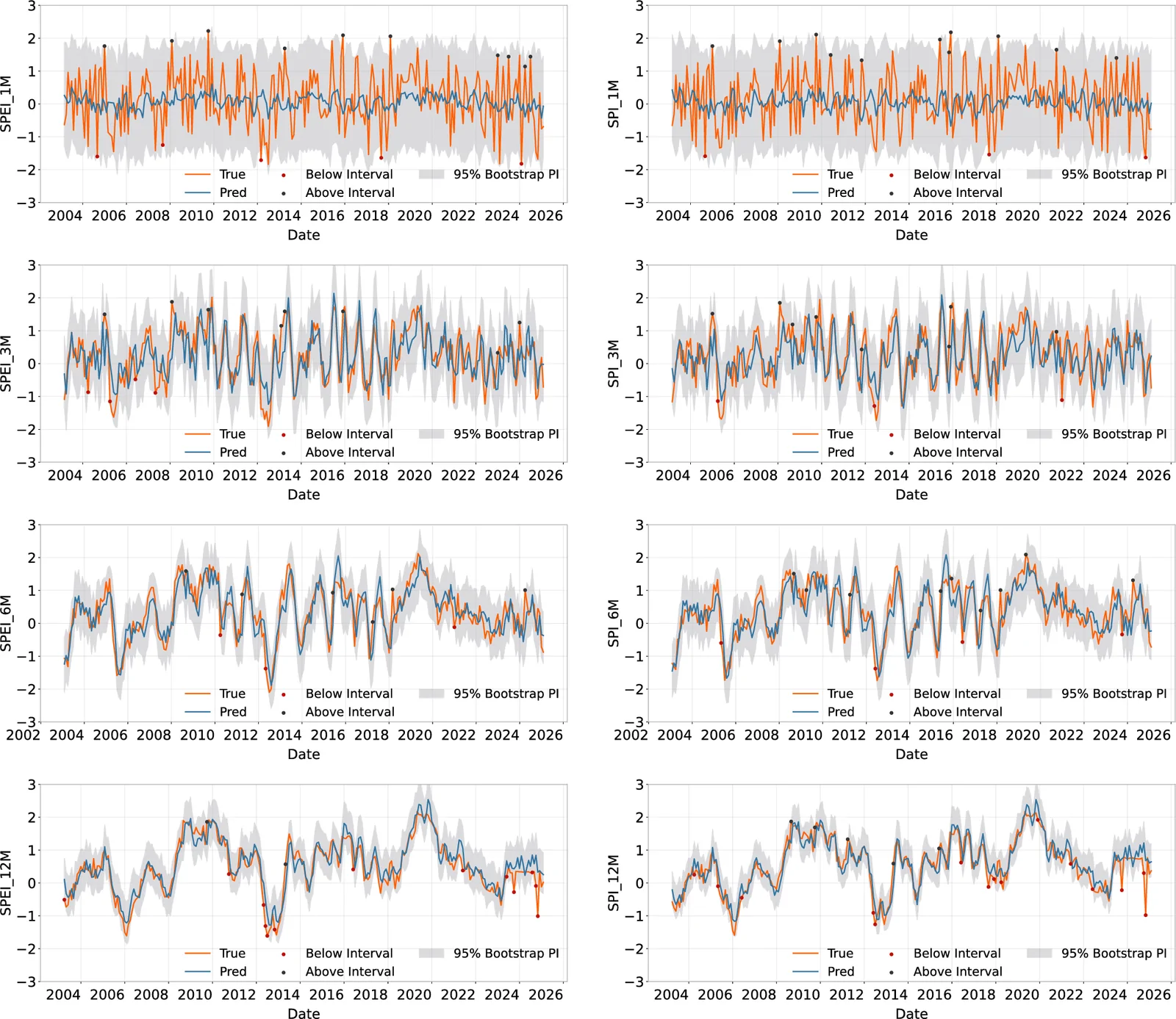
Out-of-sample predictions for SPEI (left column) and SPI (right column) across 1-, 3-, 6-, and 12-month accumulation scales (rows), showing observed and predicted drought indices together with bootstrap-derived 95% confidence intervals.

To quantify predictive uncertainty, we adopt a block bootstrap strategy that preserves the serial dependence inherent in monthly time series. Similar bootstrap-based approaches have been successfully applied to assess forecasting uncertainty in temporally correlated data^51^. Figure 6 illustrates the observed and predicted SPI and SPEI time series at multiple accumulation scales, together with the corresponding prediction intervals derived from the block bootstrap procedure. A clear scale-dependent behavior emerges: prediction intervals are widest at the shortest accumulation scale (1M), where forecasts exhibit higher variability and lower accuracy, and progressively narrow as the accumulation period increases to 3M, 6M, and 12M, which is in line with the results presented in^52^. This reduction in uncertainty reflects the increasing smoothness of the aggregated drought indices, which attenuates high-frequency variability and enhances the predictability of the underlying signal. Consequently, forecasts at longer accumulation scales, particularly at 12M, display substantially improved accuracy and more concentrated uncertainty bounds.

This result is consistent with what has been presented in several drought forecasting studies that avoid the use of one-month accumulation indices^53–55^, which may be related to the fact that, at this accumulation level, the presence of high-frequency meteorological variability tends to produce a low forecasting capacity compared to larger accumulation scales.

### Comparative benchmarking through performance metrics

**Figure 7:**
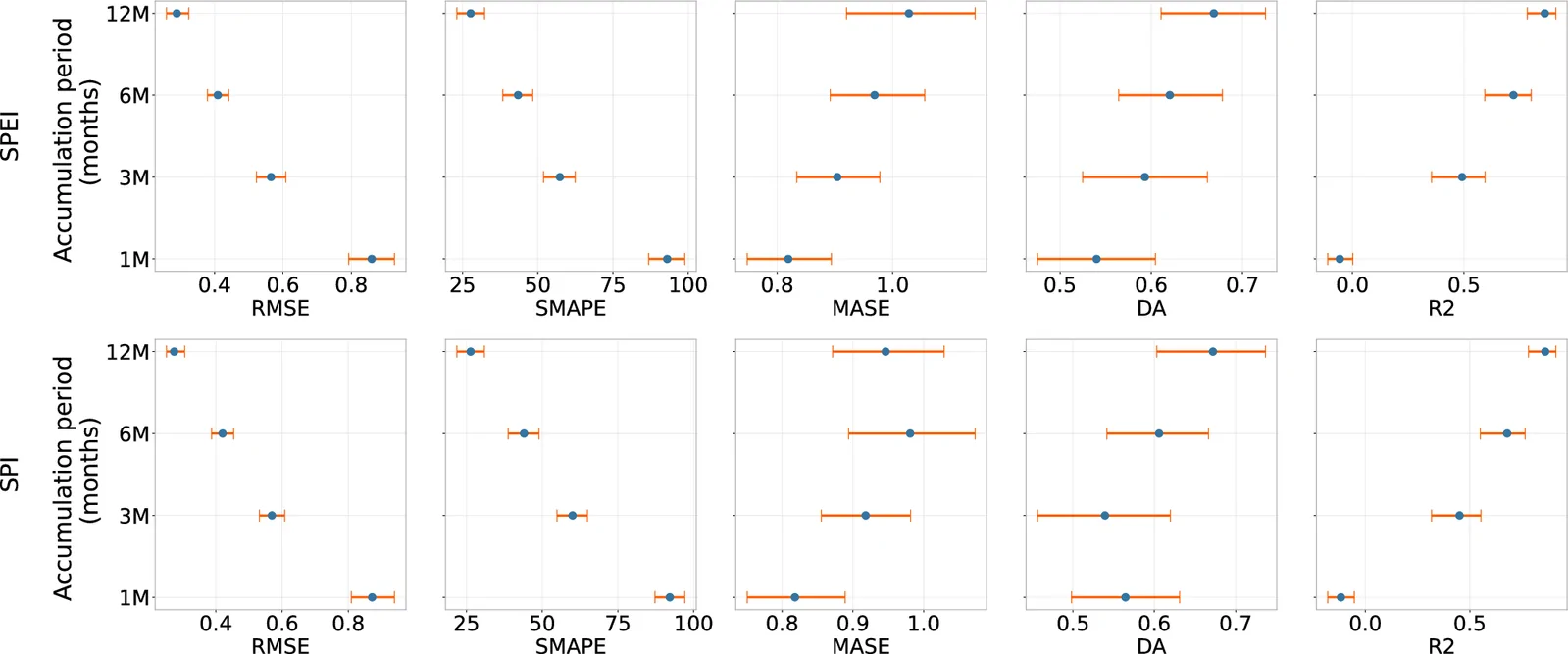
Testing performance metrics with 95% confidence intervals for SPEI (top row) and SPI (bottom row) across multiple accumulation periods: RMSE (first column), SMAPE (second column), MASE (third column), DA (fourth column) and $R^{2}$ (fifth column).

Figure 7 illustrates the testing performance metrics for SPI and SPEI across multiple time scales. Each subpanel shows the mean estimate (dot) and 95% confidence interval (horizontal line) for RMSE, SMAPE, MASE, DA, and $R^{2}$, providing a comparative assessment of model accuracy and reliability.

**Figure 8:**
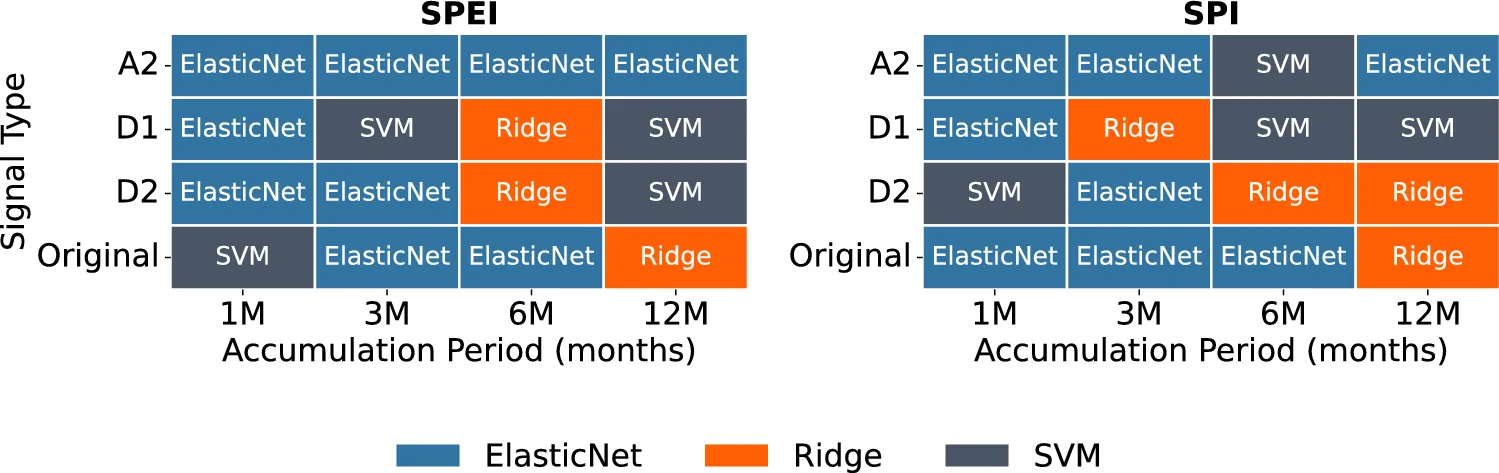
Model selection by signal for SPEI (left column) and SPI (right column) across different accumulation periods and wavelet components.

Figure 8 presents the distribution of selected models for each experimental configuration, considering both causal SWT-decomposed signals (A2, D2, D1) and the original time series without decomposition, evaluated across four accumulation periods for the SPEI and SPI indices. The results reveal a clear predominance of ElasticNet, which was selected in 53.1% of cases (17 out of 32 configurations), followed by SVM with 25.0% (8 cases) and Ridge with 21.9% (7 cases). Although additional models such as LSTM, Naïve, and HistGBM were initially evaluated, it was the regularized linear regression methods (ElasticNet and Ridge) along with SVM that frequently achieved the strongest performance metrics. The homogeneous distribution of ElasticNet across both indices (9 selections for SPEI and 8 for SPI) suggests good adaptability across the evaluated target variables.

### Interpretability and multiscale predictor-target connectivity

**Figure 9:**
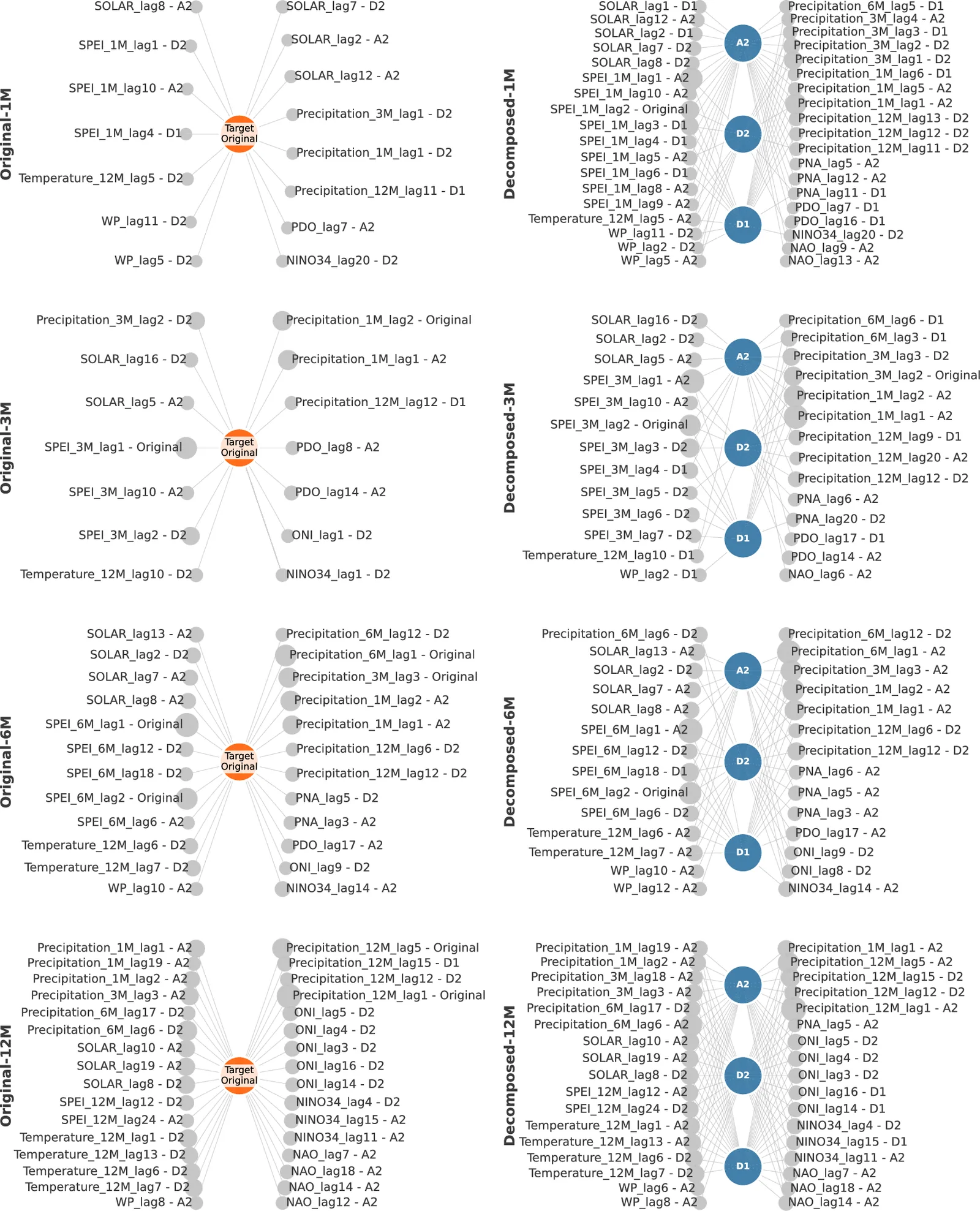
SPEI predictor networks across 1-, 3-, 6-, and 12-month accumulation periods. Orange nodes represent original series, blue nodes SWT components (A2, D2, D1), and gray nodes significant predictors. Edges denote statistically significant predictor–target relationships, while node size reflects absolute Spearman correlation strength.

Figure 9 presents a multiscale network analysis of the relationship between climate predictors and the SPEI at different accumulation periods (the corresponding results for the SPI index are provided in Appendix Fig. 11, as they follow a similar and consistent pattern). The first column displays the networks for the original SPEI series (orange nodes), while the second column illustrates the connectivity for the decomposed targets obtained through causal SWT (blue nodes: A2, D2, D1). Gray nodes represent climate variables with statistically significant Spearman correlations, with node size proportional to the absolute correlation strength. Edges represent statistically significant relationships between climate predictors and the target indices, with their weights corresponding to the absolute value of the Spearman correlation coefficient. A detailed inspection of the network density reveals that causal SWT decomposition can be interpreted as an information retrieval mechanism, substantially enhancing the detection of hydroclimatic drivers compared to the original, unprocessed series. This effect is particularly pronounced for short-term drought indices (1M and 3M), which are dominated by high-frequency variability, stronger stationarity, and reduced predictability. For SPEI_1M, the original series identifies only 15 significant predictors, whereas the decomposed representation reveals 37 unique climate predictors, corresponding to a 146% increase in predictor diversity. Importantly, these predictors establish multiple significant connections across the decomposed components: 34 predictors are associated with the A2 component, while 16 and 21 predictors are linked to the D2 and D1 components, respectively. When each predictor–component association is considered independently, this results in a total of 71 statistically significant relationships, highlighting a substantial increase in network connectivity beyond what is captured by the raw series alone. A similar amplification of connectivity is observed for SPEI-_3M, where the number of significant associations nearly doubles relative to the original series.

As the accumulation period increases to 6 and 12 months, the underlying signals become progressively smoother and more seasonal, naturally reducing the contrast between the original and decomposed representations in terms of predictor diversity. For example, the number of unique predictors increases only modestly from 24 to 28 for SPEI_6M and from 32 to 34 for SPEI_12M. However, despite this limited growth in predictor count, network connectivity continues to increase substantially. Specifically, the decomposed SPEI_6M and SPEI_12M networks exhibit 62 and 96 statistically significant edges, respectively. This reflects the fact that many predictors establish simultaneous relationships with multiple SWT components (A2, D2, and D1), revealing multiscale interactions that remain collapsed into a single averaged signal in the original series. Consequently, while long-term drought indices already capture large-scale climate forcing in their raw form, the SWT decomposition helps characterize how these climate predictors are associated across different temporal scales, leading to a denser and more informative network structure. This multiscale connectivity provides a potentially richer and more granular feature space for subsequent forecasting models, even when the set of contributing predictors remains relatively stable.

## Discussion and comparative analysis

**Figure 10:**
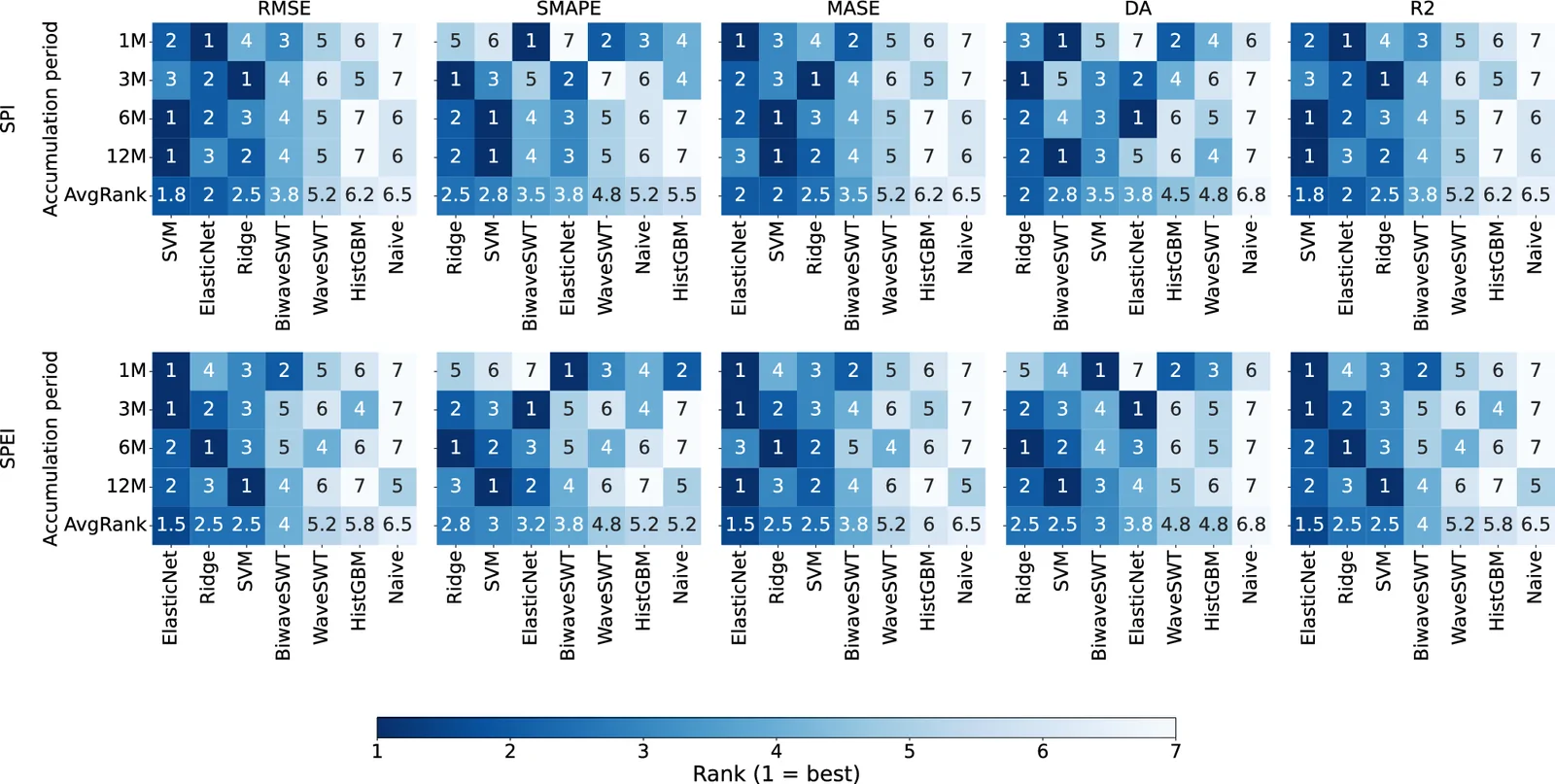
Testing ranks metrics for SPEI (top row) and SPI (bottom row) across multiple accumulation periods: RMSE (first column), SMAPE (second column), MASE (third column), DA (fourth column) and $R^{2}$ (fifth column).

Figure 10 summarizes the relative performance of the evaluated forecasting models through rank-based comparisons across multiple accumulation periods and performance metrics (Table 8 and Table 9). The DWT approaches (BiwaveDWT and WaveDWT) exhibit information leakage because wavelet decomposition implicitly uses information from the full time span when constructing transformed predictors/targets. For this reason, these two methods are not included in the main comparative plots, as their results would be artificially inflated and visually dominate the performance scale. Nevertheless, they are reported in the appendix table to explicitly illustrate how leakage can produce abnormally optimistic metrics. Overall, the proposed BiwaveSWT approach exhibits a consistently competitive behavior, most frequently ranking between third and fourth position among the seven algorithms considered for both SPI and SPEI indices.

One relevant observation is that BiwaveSWT’s relative efficiency increases as the accumulation period decreases, reaching its peak performance at the 1-month (1M) scale. This behavior is highly consistent with the network analysis presented (Figure 9), where the multiresolution decomposition was shown to uncover a substantially larger set of significant predictors for short-term indices. By successfully retrieving these ’hidden’ hydroclimatic drivers, the model gains a comparative advantage in highly volatile scenarios (1M and 3M) where standard algorithms may struggle to capture part of the underlying complexity.

Notable differences emerge when comparing rankings across evaluation metrics. Error-based measures such as RMSE, SMAPE, and MASE tend to favor models optimized for minimizing pointwise deviations, leading to slightly lower rankings for BiwaveSWT in some accumulation periods. In contrast, BiwaveSWT achieves comparatively stronger performance under the Directional Accuracy (DA) metric, where it consistently ranks among the top-performing models. This result indicates that the proposed method is particularly effective at capturing the temporal direction of drought evolution, even when absolute error magnitudes are not minimized.

The divergence between DA and error-based metrics reflects the methodological characteristics of the proposed framework. By decomposing both predictors and targets into frequency-specific components, BiwaveSWT emphasizes the preservation of phase and trend information across scales, which directly benefits directional consistency.

Overall, while the proposed approach does not systematically achieve the top rank across all error metrics, its balanced performance and strong directional skill make it a competitive and reliable alternative, particularly in applications where correctly anticipating drought onset and recovery is as critical as minimizing numerical forecast errors.

Interestingly, regularized linear models such as Ridge and ElasticNet frequently achieved strong predictive performance across multiple drought configurations and accumulation periods, occasionally outperforming the proposed BiwaveSWT framework in error-based metrics. This behavior suggests that a substantial portion of drought variability can still be effectively captured through relatively simple linear relationships when robust regularization is applied to correlated hydroclimatic predictors. In contrast, baseline approaches such as Naïve persistence and HistGBM generally exhibited lower and less stable performance across scenarios, particularly at shorter accumulation scales where signal volatility is higher. These results indicate that the proposed methodology appears to offers its greatest comparative advantage under highly dynamic multiscale conditions, while also highlighting the continued competitiveness of well-regularized statistical learning models in drought forecasting applications.

## Conclusions

This study presents a forecasting methodology for drought analysis that explicitly addresses temporal data leakage while enabling a multiscale representation of hydroclimatic time series. By applying Stationary Wavelet Transform (SWT) decomposition to both predictor variables and target drought indices, the proposed methodology extracts frequency-specific components that are subsequently modeled using machine learning algorithms. Mother wavelets are selected using an energy-based criterion to maximize variance preservation across decomposition levels. The framework was evaluated using SPI and SPEI indices at multiple accumulation scales (1-, 3-, 6-, and 12-month) over a 75-year climate record, incorporating lagged large-scale teleconnection patterns and local hydrometeorological variables. The results indicate that the proposed approach achieves stable and competitive predictive performance relative to traditional regression and ensemble-based models. The proposed model consistently outperforms naïve and univariate wavelet baselines, achieving improvements of up to 26.5% in RMSE and 4.95% in SMAPE, while also demonstrating higher directional accuracy across diverse scenarios compared to advanced methods. Crucially, the methodology demonstrates its maximum relative efficiency at shorter accumulation scales (e.g., 1-month), where high-frequency noise typically limits the performance of standard algorithms. A key finding is that the simultaneous decomposition of both predictors and targets acts as an information retrieval mechanism, uncovering significant hydroclimatic relationships that remain obscured in unprocessed time series. By isolating scale-specific components on both sides of the model, the framework identifies a richer set of statistically relevant predictors, particularly for highly volatile signals. This dual-decomposition approach not only improves forecast robustness but also provides additional insights into how climate oscillations propagate across different time–frequency domains.

Despite these promising results, several limitations should be acknowledged. The proposed framework involves a computationally intensive multiscale preprocessing pipeline, particularly when evaluating multiple decomposition levels and predictor combinations. In addition, the sample size available was restrictive for training more data-intensive deep learning architectures. Finally, the current implementation focuses on temporal dependencies and does not explicitly incorporate spatially distributed information, which may also influence drought propagation dynamics.

Future research may extend this framework by incorporating causal inference techniques, such as Convergent Cross Mapping (CCM), to identify lagged causal relationships between climate predictors and drought dynamics across temporal scales. In addition, the use of longer historical records could enable the integration of more advanced deep learning architectures, including Bidirectional LSTM (BiLSTM) and Transformer-based models, capable of capturing more complex temporal dependencies. Finally, incorporating spatial information through gridded datasets or spatially distributed drought indices represents a promising direction to further enhance the representation of spatiotemporal interactions underlying drought evolution.

## Figures and Tables

**Figure 11:**
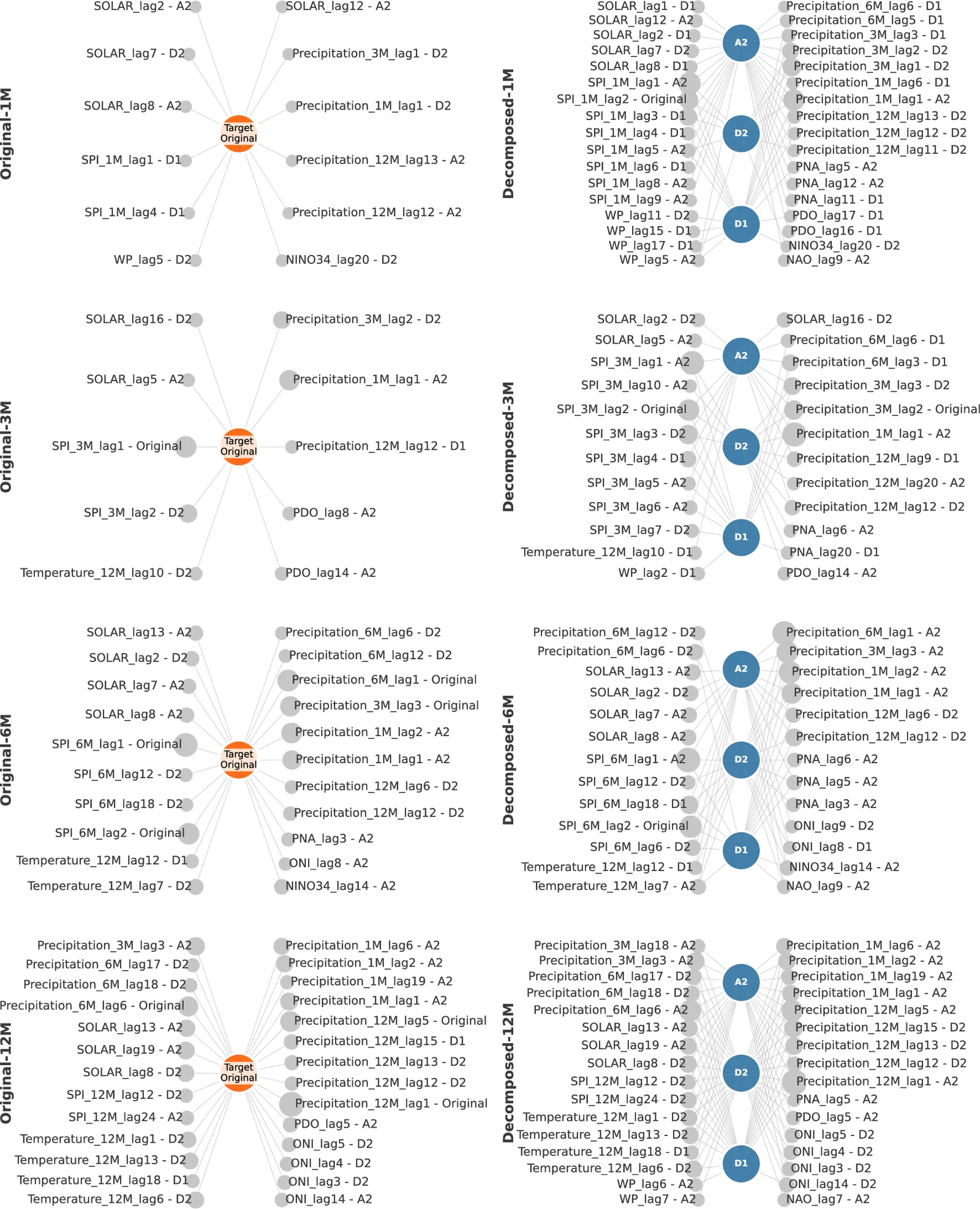
SPI predictor networks across 1-, 3-, 6-, and 12-month accumulation periods. Orange nodes represent original series, blue nodes SWT components (A2, D2, D1), and gray nodes significant predictors. Edges denote statistically significant predictor–target relationships, while node size reflects absolute Spearman correlation strength.

## Data Availability

The drought and climate datasets used in this study originate from publicly available sources cited in the manuscript. Processed datasets are stored in a GitLab repository (https://gitlab.com/elianavivas/biwave/-/tree/main) .

## Appendix A: Performance comparison of Drought forecasting models

See Tables 8, 9, 10, 11.

**Table 8:** TEST: Performance comparison of SPI forecasting models across data-leakage, proposed, and traditional approaches, including 95% confidence intervals .

Metric	Data leakage		Proposed	Traditional method					
	BiwaveDWT	WaveDWT	BiwaveSWT	WaveSWT	Ridge	ElasticNet	SVM	HistGBM	Naïve
SPI_1M-TEST									
RMSE	0.29	0.52	0.87	0.89	0.87	**0.85**	0.87	0.90	1.18
CI_low	0.26	0.45	0.81	0.83	0.82	0.80	0.81	0.84	1.07
CI_high	0.32	0.59	0.94	0.95	0.93	0.91	0.93	0.97	1.29
SMAPE	30.18	51.41	**92.13**	95.47	99.00	101.21	99.55	97.47	96.41
CI_low	27.13	45.18	87.25	89.86	94.28	96.67	95.28	92.33	88.63
CI_high	33.45	57.93	97.10	101.62	104.25	105.87	104.27	102.66	104.06
MASE	0.26	0.47	**0.82**	0.84	0.83	0.81	0.83	0.86	1.10
CI_low	0.23	0.40	0.75	0.77	0.77	0.75	0.77	0.80	0.98
CI_high	0.29	0.54	0.89	0.91	0.90	0.88	0.89	0.94	1.22
R2	0.87	0.60	-0.12	-0.16	-0.12	**-0.07**	-0.11	-0.20	-1.04
CI_low	0.85	0.51	-0.18	-0.24	-0.21	-0.15	-0.20	-0.34	-1.32
CI_high	0.89	0.68	-0.05	-0.08	-0.05	-0.02	-0.04	-0.09	-0.76
DA	0.87	0.74	**0.56**	0.42	0.42	0.00	0.41	0.48	0.35
CI_low	0.83	0.68	0.50	0.36	0.35	0.00	0.34	0.42	0.30
CI_high	0.91	0.79	0.63	0.48	0.49	0.00	0.48	0.54	0.40
SPI_3M-TEST									
RMSE	0.16	0.29	0.57	0.61	**0.52**	0.52	0.54	0.57	0.65
CI_low	0.15	0.27	0.53	0.57	0.48	0.48	0.49	0.54	0.60
CI_high	0.19	0.33	0.61	0.65	0.55	0.56	0.58	0.61	0.70
SMAPE	17.73	31.02	60.01	65.02	**52.33**	52.67	53.67	57.79	63.57
CI_low	16.12	27.02	54.84	60.36	47.88	48.26	49.52	54.23	56.80
CI_high	19.48	35.05	64.91	69.43	56.59	56.78	57.88	61.50	69.85
MASE	0.26	0.46	0.92	1.01	**0.81**	0.81	0.83	0.93	1.06
CI_low	0.24	0.41	0.86	0.93	0.75	0.75	0.77	0.86	0.96
CI_high	0.29	0.50	0.98	1.08	0.88	0.88	0.90	1.00	1.15
R2	0.95	0.85	0.45	0.36	**0.55**	0.54	0.51	0.44	0.28
CI_low	0.94	0.79	0.32	0.22	0.42	0.41	0.37	0.31	0.09
CI_high	0.97	0.90	0.55	0.47	0.65	0.65	0.63	0.55	0.43
DA	0.91	0.74	0.54	0.48	**0.64**	0.63	0.62	0.59	0.47
CI_low	0.87	0.68	0.46	0.41	0.59	0.57	0.57	0.51	0.39
CI_high	0.95	0.80	0.62	0.55	0.69	0.68	0.68	0.66	0.54
SPI_6M-TEST									
RMSE	0.19	0.27	0.42	0.43	0.38	0.38	**0.37**	0.49	0.46
CI_low	0.17	0.25	0.39	0.39	0.35	0.34	0.34	0.44	0.42
CI_high	0.21	0.30	0.45	0.47	0.41	0.41	0.41	0.54	0.50
SMAPE	19.66	30.19	43.98	44.63	38.45	38.46	**38.37**	48.23	45.51
CI_low	16.73	26.50	38.74	40.78	33.38	33.51	33.48	42.78	40.31
CI_high	22.73	33.87	48.95	48.38	43.59	43.57	43.43	53.76	50.30
MASE	0.43	0.63	0.98	1.00	0.86	0.85	**0.85**	1.12	1.05
CI_low	0.38	0.58	0.89	0.90	0.78	0.77	0.77	1.00	0.96
CI_high	0.49	0.69	1.07	1.09	0.94	0.93	0.92	1.24	1.14
R2	0.94	0.86	0.68	0.67	0.74	0.74	**0.74**	0.56	0.61
CI_low	0.91	0.80	0.55	0.52	0.62	0.62	0.63	0.36	0.45
CI_high	0.95	0.91	0.77	0.76	0.82	0.82	0.82	0.69	0.72
DA	0.84	0.70	0.61	0.57	0.65	**0.65**	0.64	0.56	0.48
CI_low	0.79	0.65	0.54	0.51	0.59	0.59	0.59	0.51	0.41
CI_high	0.88	0.75	0.67	0.63	0.71	0.71	0.70	0.62	0.55
SPI_12M-TEST									
RMSE	0.18	0.25	0.27	0.28	0.26	0.27	**0.26**	0.33	0.32
CI_low	0.15	0.22	0.25	0.25	0.24	0.24	0.23	0.30	0.28
CI_high	0.24	0.29	0.31	0.32	0.29	0.30	0.29	0.36	0.35
SMAPE	16.25	22.86	26.34	27.12	24.35	24.82	**23.76**	29.76	29.13
CI_low	12.60	18.45	21.80	22.77	19.89	20.51	19.37	25.29	23.76
CI_high	20.18	27.71	30.88	31.29	28.66	28.80	27.94	34.16	34.17
MASE	0.60	0.85	0.95	0.99	0.89	0.89	**0.87**	1.11	1.05
CI_low	0.50	0.74	0.87	0.90	0.80	0.81	0.77	0.99	0.93
CI_high	0.71	0.96	1.03	1.08	0.98	0.98	0.97	1.24	1.17
R2	0.94	0.88	0.86	0.85	0.87	0.87	**0.88**	0.80	0.81
CI_low	0.88	0.80	0.78	0.76	0.80	0.79	0.80	0.71	0.71
CI_high	0.97	0.93	0.91	0.91	0.92	0.92	0.93	0.86	0.89
DA	0.85	0.65	**0.67**	0.63	0.66	0.62	0.66	0.56	0.54
CI_low	0.81	0.60	0.60	0.56	0.58	0.54	0.58	0.49	0.47
CI_high	0.89	0.69	0.74	0.70	0.74	0.68	0.74	0.64	0.60

Significant values are in bold.

**Table 9:** TEST: Performance comparison of SPEI forecasting models across data-leakage, proposed, and traditional approaches, including 95% confidence intervals.

Metric	Data leakage		Proposed	Traditional method					
	BiwaveDWT	WaveDWT	BiwaveSWT	WaveSWT	Ridge	ElasticNet	SVM	HistGBM	Naïve
SPEI_1M-TEST									
RMSE	0.26	0.56	**0.86**	0.87	0.87	0.86	0.86	0.91	1.17
CI_low	0.23	0.48	0.79	0.81	0.81	0.80	0.80	0.85	1.06
CI_high	0.29	0.63	0.93	0.93	0.93	0.91	0.92	0.97	1.29
SMAPE	27.72	55.59	**93.04**	97.89	98.67	100.24	99.95	97.95	95.17
CI_low	25.01	49.31	86.90	92.69	93.97	95.84	95.29	92.22	87.33
CI_high	30.58	62.41	98.90	103.10	103.30	104.50	104.50	103.32	103.53
MASE	0.24	0.51	0.82	0.83	0.83	**0.82**	0.82	0.87	1.09
CI_low	0.22	0.43	0.75	0.77	0.77	0.75	0.76	0.80	0.98
CI_high	0.27	0.58	0.89	0.90	0.90	0.88	0.89	0.95	1.22
R2	0.90	0.55	-0.06	-0.09	-0.08	**-0.05**	-0.06	-0.19	-0.97
CI_low	0.88	0.46	-0.11	-0.18	-0.16	-0.12	-0.14	-0.32	-1.25
CI_high	0.92	0.64	0.00	-0.02	-0.01	0.00	-0.01	-0.08	-0.69
DA	0.88	0.73	**0.54**	0.44	0.38	0.33	0.42	0.43	0.35
CI_low	0.84	0.68	0.48	0.37	0.32	0.29	0.35	0.37	0.29
CI_high	0.92	0.79	0.60	0.52	0.45	0.38	0.48	0.49	0.40
SPEI_3M-TEST									
RMSE	0.17	0.32	0.57	0.59	0.51	**0.51**	0.52	0.56	0.65
CI_low	0.15	0.29	0.52	0.54	0.47	0.47	0.48	0.52	0.60
CI_high	0.20	0.35	0.61	0.63	0.55	0.54	0.56	0.60	0.70
SMAPE	17.99	32.29	57.34	59.77	50.83	**50.45**	51.50	56.35	61.08
CI_low	16.06	28.50	51.89	55.23	46.74	46.21	47.09	53.28	54.87
CI_high	19.95	36.40	62.41	64.45	54.92	54.59	55.71	59.75	67.20
MASE	0.27	0.49	0.90	0.95	0.80	**0.80**	0.82	0.91	1.03
CI_low	0.24	0.45	0.83	0.87	0.74	0.74	0.75	0.84	0.94
CI_high	0.30	0.53	0.98	1.04	0.87	0.86	0.88	0.98	1.12
R2	0.95	0.84	0.49	0.45	0.58	**0.59**	0.57	0.51	0.33
CI_low	0.94	0.78	0.35	0.35	0.47	0.47	0.44	0.39	0.15
CI_high	0.97	0.88	0.59	0.53	0.67	0.68	0.67	0.60	0.48
DA	0.92	0.71	0.59	0.54	0.61	**0.61**	0.61	0.56	0.45
CI_low	0.89	0.64	0.52	0.48	0.54	0.55	0.56	0.49	0.38
CI_high	0.95	0.77	0.66	0.59	0.67	0.67	0.66	0.63	0.51
SPEI_6M-TEST									
RMSE	0.16	0.28	0.41	0.41	**0.36**	0.36	0.37	0.45	0.45
CI_low	0.14	0.25	0.38	0.37	0.33	0.34	0.34	0.41	0.41
CI_high	0.18	0.30	0.44	0.44	0.39	0.39	0.39	0.49	0.49
SMAPE	17.69	29.90	43.43	41.41	**37.33**	38.16	37.73	45.70	45.95
CI_low	15.22	25.88	38.32	37.07	32.81	33.71	33.51	40.89	40.50
CI_high	20.18	34.14	48.35	45.83	42.06	42.83	42.24	50.31	51.29
MASE	0.37	0.63	0.97	0.92	0.83	0.84	**0.83**	1.04	1.05
CI_low	0.33	0.57	0.89	0.83	0.76	0.77	0.76	0.95	0.96
CI_high	0.41	0.68	1.06	1.02	0.90	0.91	0.90	1.15	1.14
R2	0.96	0.87	0.72	0.73	**0.79**	0.78	0.78	0.67	0.66
CI_low	0.94	0.80	0.59	0.61	0.70	0.69	0.69	0.54	0.49
CI_high	0.97	0.92	0.80	0.81	0.84	0.84	0.84	0.76	0.77
DA	0.88	0.71	0.62	0.58	**0.68**	0.67	0.67	0.60	0.50
CI_low	0.84	0.66	0.56	0.52	0.63	0.61	0.62	0.55	0.43
CI_high	0.91	0.75	0.68	0.64	0.73	0.72	0.72	0.67	0.57
SPEI_12M-TEST									
RMSE	0.22	0.26	0.29	0.33	0.28	0.27	**0.27**	0.39	0.30
CI_low	0.18	0.22	0.26	0.30	0.25	0.25	0.24	0.34	0.27
CI_high	0.26	0.30	0.32	0.37	0.31	0.31	0.30	0.44	0.33
SMAPE	20.90	25.00	27.68	32.54	26.40	26.11	**25.74**	34.50	29.11
CI_low	16.84	20.14	23.08	26.88	21.75	21.82	21.27	30.15	24.13
CI_high	25.14	30.10	32.28	38.05	31.05	30.26	30.47	38.63	33.78
MASE	0.79	0.93	1.03	1.20	1.02	**0.97**	0.98	1.38	1.05
CI_low	0.66	0.80	0.92	1.08	0.90	0.87	0.87	1.20	0.93
CI_high	0.93	1.07	1.14	1.34	1.14	1.07	1.10	1.58	1.17
R2	0.92	0.89	0.86	0.82	0.87	**0.88**	0.88	0.75	0.85
CI_low	0.85	0.81	0.78	0.70	0.78	0.80	0.79	0.65	0.76
CI_high	0.96	0.94	0.91	0.89	0.93	0.92	0.93	0.83	0.91
DA	0.81	0.64	0.67	0.62	0.67	0.64	**0.68**	0.58	0.54
CI_low	0.75	0.59	0.61	0.55	0.59	0.57	0.61	0.50	0.47
CI_high	0.85	0.70	0.73	0.69	0.74	0.70	0.75	0.66	0.61

Significant values are in bold.

**Table 10:** TRAIN: Performance comparison of SPEI predicting models across data-leakage, proposed, and traditional approaches, including 95% confidence intervals.

Metric	Data leakage		Proposed	Traditional method					
	BiwaveDWT	WaveDWT	BiwaveSWT	WaveSWT	Ridge	ElasticNet	SVM	HistGBM	Naïve
SPEI_1M-TRAIN									
RMSE	0.22	0.48	0.80	0.78	0.79	0.81	0.75	0.48	1.07
CI_low	0.21	0.44	0.75	0.74	0.75	0.76	0.71	0.46	1.00
CI_high	0.24	0.52	0.84	0.82	0.83	0.85	0.79	0.51	1.13
SMAPE	25.04	50.88	87.71	86.46	90.53	95.20	83.81	49.28	92.34
CI_low	23.46	47.75	84.07	82.81	87.07	91.85	80.29	47.08	88.17
CI_high	26.62	54.25	91.20	90.03	93.95	98.60	87.26	51.39	96.48
MASE	0.21	0.44	0.75	0.73	0.74	0.75	0.69	0.45	1.00
CI_low	0.19	0.41	0.70	0.68	0.70	0.71	0.65	0.42	0.93
CI_high	0.22	0.48	0.79	0.77	0.78	0.80	0.73	0.48	1.07
R2	0.92	0.65	0.03	0.08	0.04	0.01	0.14	0.64	-0.75
CI_low	0.91	0.59	-0.01	0.05	0.02	0.00	0.11	0.62	-0.90
CI_high	0.93	0.70	0.06	0.10	0.06	0.01	0.16	0.66	-0.60
DA	0.90	0.78	0.55	0.50	0.49	0.37	0.76	0.88	0.37
CI_low	0.88	0.75	0.51	0.45	0.45	0.33	0.72	0.85	0.34
CI_high	0.92	0.82	0.59	0.55	0.53	0.41	0.80	0.91	0.40
SPEI_3M-TRAIN									
RMSE	0.15	0.28	0.52	0.57	0.47	0.46	0.47	0.33	0.64
CI_low	0.14	0.27	0.49	0.54	0.44	0.44	0.44	0.31	0.60
CI_high	0.16	0.30	0.55	0.60	0.49	0.49	0.49	0.36	0.68
SMAPE	17.00	32.19	54.74	60.42	49.64	49.33	48.92	35.73	60.63
CI_low	15.79	30.08	51.56	57.83	47.29	46.96	46.77	33.64	57.73
CI_high	18.26	34.63	57.74	63.02	51.91	51.64	51.21	37.64	63.74
MASE	0.24	0.46	0.84	0.93	0.75	0.75	0.74	0.54	1.00
CI_low	0.22	0.43	0.79	0.87	0.71	0.70	0.70	0.50	0.94
CI_high	0.26	0.49	0.89	0.98	0.79	0.79	0.78	0.57	1.07
R2	0.97	0.88	0.60	0.52	0.67	0.68	0.67	0.83	0.39
CI_low	0.96	0.86	0.55	0.47	0.63	0.64	0.63	0.81	0.31
CI_high	0.97	0.90	0.65	0.56	0.71	0.71	0.71	0.85	0.47
DA	0.92	0.77	0.63	0.59	0.65	0.66	0.66	0.77	0.54
CI_low	0.89	0.73	0.58	0.55	0.61	0.62	0.62	0.73	0.50
CI_high	0.94	0.80	0.67	0.63	0.69	0.70	0.70	0.80	0.59
SPEI_6M-TRAIN									
RMSE	0.14	0.27	0.38	0.37	0.32	0.32	0.32	0.11	0.45
CI_low	0.13	0.25	0.36	0.35	0.30	0.30	0.30	0.10	0.42
CI_high	0.16	0.29	0.40	0.39	0.33	0.34	0.34	0.11	0.48
SMAPE	15.67	29.35	40.18	38.81	33.84	33.97	34.19	11.34	43.59
CI_low	14.23	27.01	37.83	35.95	31.75	31.86	31.97	10.45	40.36
CI_high	17.24	31.66	42.66	41.78	36.03	36.22	36.56	12.33	46.58
MASE	0.33	0.63	0.88	0.85	0.73	0.73	0.73	0.24	1.00
CI_low	0.30	0.59	0.83	0.79	0.69	0.69	0.69	0.23	0.94
CI_high	0.35	0.67	0.93	0.90	0.77	0.78	0.78	0.26	1.07
R2	0.97	0.89	0.78	0.79	0.84	0.84	0.84	0.98	0.69
CI_low	0.96	0.86	0.74	0.75	0.82	0.81	0.81	0.98	0.62
CI_high	0.97	0.91	0.82	0.83	0.87	0.87	0.87	0.99	0.75
DA	0.84	0.70	0.65	0.64	0.67	0.67	0.69	0.88	0.53
CI_low	0.82	0.66	0.61	0.60	0.64	0.63	0.66	0.86	0.50
CI_high	0.87	0.73	0.68	0.68	0.71	0.71	0.72	0.90	0.57
SPEI_12M-TRAIN									
RMSE	0.18	0.24	0.26	0.28	0.24	0.26	0.25	0.17	0.28
CI_low	0.17	0.22	0.24	0.26	0.23	0.24	0.23	0.16	0.27
CI_high	0.20	0.26	0.28	0.29	0.26	0.28	0.27	0.19	0.30
SMAPE	20.12	25.80	28.28	30.29	26.55	28.34	26.37	17.81	30.00
CI_low	17.52	22.80	25.36	26.99	24.02	25.71	23.88	16.08	26.89
CI_high	23.35	29.42	31.68	33.92	29.30	31.26	29.11	19.78	33.08
MASE	0.66	0.86	0.94	1.01	0.90	0.95	0.90	0.60	1.00
CI_low	0.59	0.79	0.87	0.93	0.83	0.88	0.83	0.55	0.94
CI_high	0.74	0.93	1.01	1.08	0.96	1.02	0.96	0.65	1.07
R2	0.95	0.91	0.89	0.88	0.90	0.89	0.90	0.95	0.87
CI_low	0.92	0.87	0.85	0.83	0.87	0.85	0.87	0.94	0.82
CI_high	0.96	0.93	0.92	0.91	0.93	0.92	0.93	0.96	0.91
DA	0.81	0.68	0.65	0.63	0.67	0.64	0.67	0.76	0.57
CI_low	0.78	0.64	0.62	0.59	0.63	0.60	0.64	0.73	0.53
CI_high	0.84	0.71	0.69	0.67	0.70	0.68	0.71	0.79	0.60

**Table 11:** TRAIN: Performance comparison of SPI-predicting models across data-leakage, proposed, and traditional approaches, including 95% confidence intervals.

Metric	Data leakage		Proposed	Traditional method					
	BiwaveDWT	WaveDWT	BiwaveSWT	WaveSWT	Ridge	ElasticNet	SVM	HistGBM	Naïve
SPI_1M-TRAIN									
RMSE	0.25	0.48	0.80	0.78	0.79	0.81	0.80	0.39	1.08
CI_low	0.23	0.44	0.75	0.74	0.75	0.77	0.76	0.37	1.02
CI_high	0.26	0.52	0.84	0.82	0.83	0.85	0.85	0.41	1.15
SMAPE	26.99	50.17	87.67	87.58	91.83	96.88	92.85	37.58	92.08
CI_low	24.90	46.91	84.01	84.51	88.26	93.07	89.00	35.97	87.81
CI_high	28.99	53.69	91.30	90.69	95.13	100.55	96.51	39.24	96.33
MASE	0.23	0.44	0.75	0.73	0.75	0.76	0.75	0.36	1.00
CI_low	0.21	0.40	0.70	0.69	0.70	0.71	0.71	0.34	0.94
CI_high	0.24	0.47	0.79	0.78	0.79	0.81	0.80	0.38	1.07
R2	0.91	0.65	0.02	0.07	0.04	0.00	0.01	0.77	-0.80
CI_low	0.90	0.59	-0.02	0.03	0.02	-0.01	-0.01	0.75	-0.96
CI_high	0.92	0.71	0.06	0.10	0.05	0.00	0.02	0.78	-0.65
MDA	0.90	0.76	0.56	0.49	0.48	0.00	0.48	0.90	0.36
CI_low	0.87	0.72	0.52	0.46	0.44	0.00	0.44	0.88	0.32
CI_high	0.92	0.79	0.60	0.53	0.53	0.01	0.53	0.92	0.40
	SPI_3M-TRAIN								
RMSE	0.14	0.27	0.54	0.58	0.47	0.47	0.48	0.34	0.64
CI_low	0.13	0.25	0.51	0.54	0.45	0.45	0.45	0.31	0.60
CI_high	0.15	0.29	0.56	0.61	0.50	0.50	0.51	0.36	0.68
SMAPE	15.53	30.48	58.14	60.21	49.64	49.79	48.44	35.99	61.32
CI_low	14.55	28.33	55.33	57.53	47.43	47.51	46.03	33.70	58.46
CI_high	16.61	32.85	60.88	62.79	51.87	52.06	50.84	38.12	64.31
MASE	0.22	0.43	0.87	0.92	0.75	0.75	0.74	0.53	1.00
CI_low	0.20	0.40	0.82	0.86	0.70	0.70	0.69	0.50	0.94
CI_high	0.23	0.46	0.92	0.98	0.79	0.79	0.79	0.57	1.07
R2	0.97	0.89	0.56	0.49	0.66	0.66	0.65	0.83	0.37
CI_low	0.97	0.87	0.51	0.44	0.61	0.61	0.60	0.81	0.28
CI_high	0.97	0.91	0.61	0.53	0.70	0.70	0.69	0.85	0.45
MDA	0.92	0.76	0.59	0.54	0.66	0.66	0.65	0.76	0.54
CI_low	0.89	0.74	0.55	0.49	0.62	0.62	0.61	0.73	0.50
CI_high	0.94	0.79	0.63	0.58	0.70	0.70	0.69	0.80	0.58
SPI_6M-TRAIN									
RMSE	0.16	0.27	0.38	0.37	0.32	0.32	0.32	0.10	0.45
CI_low	0.15	0.25	0.36	0.34	0.30	0.30	0.31	0.10	0.42
CI_high	0.17	0.29	0.40	0.39	0.34	0.34	0.34	0.11	0.49
SMAPE	17.37	29.42	40.88	38.61	34.73	34.79	34.55	11.26	43.75
CI_low	15.83	27.09	38.50	36.05	32.47	32.51	32.14	10.31	40.69
CI_high	19.03	31.91	43.58	41.36	37.05	37.11	36.94	12.25	46.85
MASE	0.36	0.62	0.87	0.83	0.74	0.74	0.74	0.23	1.00
CI_low	0.33	0.58	0.82	0.78	0.70	0.70	0.69	0.21	0.93
CI_high	0.39	0.66	0.92	0.89	0.79	0.79	0.79	0.24	1.07
R2	0.96	0.89	0.77	0.79	0.84	0.84	0.83	0.98	0.67
CI_low	0.95	0.86	0.73	0.75	0.81	0.81	0.80	0.98	0.61
CI_high	0.97	0.91	0.81	0.82	0.86	0.86	0.86	0.99	0.73
MDA	0.85	0.70	0.64	0.61	0.68	0.68	0.67	0.91	0.53
CI_low	0.82	0.66	0.60	0.57	0.65	0.65	0.64	0.89	0.49
CI_high	0.88	0.73	0.67	0.65	0.71	0.72	0.71	0.93	0.57
SPI_12M-TRAIN									
RMSE	0.13	0.21	0.24	0.26	0.22	0.24	0.22	0.07	0.30
CI_low	0.12	0.19	0.23	0.24	0.21	0.22	0.21	0.07	0.28
CI_high	0.14	0.23	0.26	0.27	0.23	0.25	0.24	0.08	0.32
SMAPE	14.22	23.28	26.17	26.98	23.55	25.58	23.78	8.05	31.28
CI_low	12.47	20.71	23.64	24.52	21.40	23.32	21.58	7.21	28.31
CI_high	15.97	26.50	28.83	29.63	25.69	27.83	25.96	9.01	34.50
MASE	0.44	0.73	0.84	0.87	0.76	0.82	0.77	0.25	1.00
CI_low	0.40	0.67	0.79	0.81	0.71	0.77	0.72	0.23	0.94
CI_high	0.47	0.80	0.90	0.93	0.81	0.87	0.82	0.26	1.07
R2	0.97	0.93	0.90	0.89	0.92	0.91	0.92	0.99	0.85
CI_low	0.96	0.90	0.87	0.85	0.89	0.88	0.89	0.99	0.80
CI_high	0.98	0.95	0.92	0.92	0.94	0.93	0.94	0.99	0.89
MDA	0.85	0.68	0.68	0.62	0.68	0.65	0.68	0.88	0.54
CI_low	0.82	0.64	0.65	0.59	0.65	0.61	0.65	0.86	0.50
CI_high	0.87	0.72	0.71	0.66	0.71	0.69	0.71	0.91	0.58

## Appendix B: Network graphs

See Fig. 11.

## Drought indices

PDSI: Palmer drought severity index
sc-PDSI: Self-calibrated palmer drought severity index
SGI: Standardized groundwater level index
SPEI: Standardized precipitation evapotranspiration index
SPI: Standardized precipitation index
SRI: Standardized runoff index

## Climate predictors

F10.7: 10.7 cm solar flux, a proxy for solar activity
NAO: North Atlantic oscillation
Niño 3.4: Niño 3.4 Index (SST anomalies in the central equatorial Pacific)
ONI: Oceanic Niño Index
PDO: Pacific decadal oscillation
PNA: Pacific–North American Index
WP: Western Pacific Index

## Time series & signal processing

ACF: Autocorrelation function
CCF: Cross-correlation function
DWT: Discrete wavelet transform
ICEEMDAN: Improved complete ensemble empirical mode decomposition with adaptive noise
IMF: Intrinsic mode function
MODWT: Maximal overlap discrete wavelet transform
PACF: Partial autocorrelation function
WD: Wavelet decomposition
sym6: Symlet wavelet with six vanishing moments
TDIC: Time-dependent intrinsic correlation
TQWT: Tunable Q-factor wavelet transform
VMD: Variation mode decomposition
WDDFF: Wavelet data-driven forecasting framework
SWT: Stationary wavelet transform

## Forecasting models

ANFIS: Adaptive neuro-fuzzy inference system
ANN: Artificial neural network
ElasticNet: Regularized linear regression combining L1 and L2 penalties
GPR: Gaussian process regression
GWO: Gray wolf optimization
HistGBM: Histogram-based gradient boosting machine
MLP: Multilayer Perceptron
MLR: Multiple linear regression
Naïve: Naïve forecasting benchmark model
PCR: Principal component regression
QRNN: Quantile Regression Neural Network
Ridge: L2-regularized linear regression
SARIMA: Seasonal autoregressive integrated moving average
SVM: Support vector machine

## Performance metrics

DA: Directional accuracy
MASE: Mean absolute scaled error
$R^2$: Coefficient of determination
RMSE: Root mean square error
SMAPE: Symmetric mean absolute percentage error

## Code availability

The code used and/or analyzed during the current study is stored in a private GitLab repository (https://gitlab.com/elianavivas/biwave) and is available upon reasonable request to the corresponding author.

## Materials availability

The materials used and/or analyzed during the current study are stored in a private GitLab repository (https://gitlab.com/elianavivas/biwave) and are available upon reasonable request to the corresponding author.

## References

[1] Naik, A. K., Ajetrao, K. V. & Nagori, N. S. *Theory of Wavelets: From Design Principles to Applications* (Techsar Pvt Ltd, India, 2024).

[2] Ramakrishnan, S. *Modern Appl. Wav. Transform* (BoD-Books on Demand, London, United Kingdom, 2024).

[3] Kim, T. W. & Valdés, J. B. Nonlinear model for drought forecasting based on a conjunction of wavelet transforms and neural networks. *J. Hydrol. Eng.* **8**(6), 319–328 (2003).

[4] Özger, M. et al. Comparison of wavelet and empirical mode decomposition hybrid models in drought prediction. *Comput. Electron. Agric.* **179**, 105851 (2020).

[5] Karbasi, M. et al. Development of a new wavelet-based hybrid model to forecast multi-scalar spei drought index (case study: Zanjan city, iran). *Theor. Appl. Climatol.* **147**(1), 499–522 (2022).

[6] Achite, M. et al. Analyzing the dynamical relationships between meteorological and hydrological drought of wadi mina basin, algeria using a novel multiscale framework. *Stoch. Environ. Res. Risk Assess.* **38**(5), 1935–1953 (2024).

[7] Tuğrul, T., Hınıs, M. A. & Oruç, S. Comparison of lstm and svm methods through wavelet decomposition in drought forecasting. *Earth Sci. Inform.* **18**(1), 139 (2025).

[8] Rezaiy, R. & Shabri, A. Integrating wavelet transform and support vector machine for improved drought forecasting based on standardized precipitation index. *J. Hydroinform.* **27**(2), 320–337 (2025).

[9] Shahnazi, S., Roushangar, K. & Hashemi, H. A novel implementation of pre-processing approaches and hybrid kernel-based model for short-and long-term groundwater drought forecasting. *J. Hydrol.* **652**, 132667 (2025).

[10] Han, J. et al. Forecasting compound drought-heatwaves using burg entropy spectral analysis with multi-frequency resolutions. *J. Hydrol.* **658**, 133166 (2025).

[11] Osmani, S. A. et al. Wavelet-based precipitation preprocessing for improved drought forecasting: A machine learning approach using tunable q-factor wavelet transform and maximal overlap discrete wavelet transform. *Expert Syst. Appl.* **257**, 124962 (2024).

[12] Waqas, M., Humphries, U. W. & Hlaing, P. T. Time series trend analysis and forecasting of climate variability using deep learning in thailand. *Res. Eng.* **24**, 102997 (2024).

[13] Vivas, E. et al. Deep lagged-wavelet for monthly rainfall forecasting in a tropical region. *Stoch. Environ. Res. Risk Assess.* **37**(3), 831–848 (2023).

[14] Ahmadi, F. et al. Prediction of precipitation using wavelet-based hybrid models considering the periodicity. *Neural Comput. Appl.* **36**(26), 16345–16364 (2024).

[15] Vivas, E. et al. Long short-term memory wavelet neural network for renewable energy generation forecasting. *Int. J. Intell. Syst.* **2025**(1), 8890906 (2025).

[16] Vivas, E., Allende-Cid, H. & Salas, R. A systematic review of statistical and machine learning methods for electrical power forecasting with reported mape score. *Entropy* **22**(12), 1412 (2020). 33333829 10.3390/e22121412 PMC7765272

[17] Sadiqi, S. S. J. et al. An integrated framework for understanding ecological drought and drought resistance. *Sci. Total Environ.* **846**, 157477 (2022). 35870577 10.1016/j.scitotenv.2022.157477

[18] Zhou, Z. et al. Recent development on drought propagation: A comprehensive review. *J. Hydrol.* **645**, 132196 (2024).

[19] Deznabi, I. & Fiterau, M. Multiwave: Multiresolution deep architectures through wavelet decomposition for multivariate timeseries forecasting and prediction (2023).

[20] Mohammadi, B. et al. Enhancing streamflow drought prediction: Integrating wavelet decomposition with deep learning and quantile regression neural network models. *Earth Sci. Inform.* **18**(2), 232 (2025).

[21] Pang, J., & Dong, S. Stop unrealistic data preprocessing in wind speed forecasting: approaches and discussions on preventing future data leakage. Applied Mathematical Modelling p 116376 (2025)

[22] Quilty, J. & Adamowski, J. Addressing the incorrect usage of wavelet-based hydrological and water resources forecasting models for real-world applications with best practices and a new forecasting framework. *J. Hydrol.* **563**, 336–353 (2018).

[23] Khan, M. M. H., Muhammad, N. S. & El-Shafie, A. Wavelet based hybrid ann-arima models for meteorological drought forecasting. *J. Hydrol.* **590**, 125380 (2020).

[24] Nwayor, I. J. & Robeson, S. M. Exploring the relationship between spi and spei in a warming world. *Theor. Appl. Climatol.* **155**(4), 2559–2569 (2024).

[25] Yusuf, B., Shabri, A. & Rezaiy, R. An innovative hybrid drought modelling approach using wavelet-long-short term memory (w-lstm) with standardised precipitation index (spi) data for badeggi district, niger state-nigeria. *Discov. Appl. Sci.* **7**(10), 1129 (2025).

[26] Serban, C., Ionescu, A. F. & Maftei, C. Drought forecasting in dobrogea, romania using spei-based machine learning models. *Stoch. Environ. Res. Risk Assess.* **40**(5), 121 (2026).

[27] Western Regional Climate Center (2024) Westwide drought tracker (wwdt). https://wrcc.dri.edu/wwdt/time/, accessed: 2025-07-28

[28] Zheng, Y. et al. Assessing the joint impact of climatic variables on meteorological drought using machine learning. *Front. Earth Sci.* **10**, 835142 (2022).

[29] Sabzevari, Y. et al. Drought trend analysis using standardized precipitation evapotranspiration index in cold-climate regions. *Atmosphere* **16**(4), 482 (2025).

[30] NOAA Physical Sciences Laboratory (2024a) Nao: Monthly atmospheric and ocean time series. https://psl.noaa.gov/data/correlation/nao.data, accessed: 2025-07-20

[31] NOAA Physical Sciences Laboratory (2024c) Oceanic niño index (oni) data. https://psl.noaa.gov/data/correlation/oni.data, accessed: 2025-07-28

[32] NOAA Physical Sciences Laboratory (2024b) North atlantic oscillation (nao) data. https://psl.noaa.gov/data/correlation/nao.data, accessed: 2025-07-28

[33] NOAA Physical Sciences Laboratory (2024d) Pacific decadal oscillation (pdo) data. https://psl.noaa.gov/pdo/data/pdo.timeseries.sstens.data, accessed: 2025-07-28

[34] NOAA Climate Prediction Center (2024) Ersstv5 niño 3.4 sst anomaly data. https://www.cpc.ncep.noaa.gov/data/indices/ersst5.nino.mth.91-20.ascii, accessed: 2025-07-28

[35] NOAA Physical Sciences Laboratory (2024f) Western pacific (wp) index data. https://psl.noaa.gov/data/correlation/wp.data, accessed: 2025-07-28

[36] NOAA Physical Sciences Laboratory (2024e) Pacific-north american (pna) index data. https://psl.noaa.gov/data/correlation/pna.data, accessed: 2025-07-28

[37] Natural Resources Canada (2024) Solar flux monthly average data. https://spaceweather.ca/forecast-prevision/solar-solaire/solarflux/sx-5-mavg-en.php, accessed: 2025-07-28

[38] Granger, C. & Newbold, P. Spurious regressions in econometrics. *J. Econom.* **2**, 111–120 (1974).

[39] Benaouda, D. et al. Wavelet-based nonlinear multiscale decomposition model for electricity load forecasting. *Neurocomputing* **70**(1–3), 139–154 (2006).

[40] Adamowski, J. & Chan, H. F. A wavelet neural network conjunction model for groundwater level forecasting. *J. Hydrol.* **407**(1–4), 28–40 (2011).

[41] Shalby, E. M. et al. A comprehensive guide to selecting suitable wavelet decomposition level and functions in discrete wavelet transform for fault detection in distribution networks. *Sci. Rep.* **15**(1), 1160 (2025). 39775005 10.1038/s41598-024-82025-2 PMC11706944

[42] Nourani, V. et al. Applications of hybrid wavelet-artificial intelligence models in hydrology: a review. *J. Hydrol.* **514**, 358–377 (2014).

[43] Fung, K. et al. Drought forecasting: A review of modelling approaches 2007–2017. *J. Water . Change* **11**(3), 771–799 (2020).

[44] Grinsted, A., Moore, J. C. & Jevrejeva, S. Application of the cross wavelet transform and wavelet coherence to geophysical time series. *Nonlinear Process. Geophys.* **11**(5/6), 561–566 (2004).

[45] Nalley, D., Adamowski, J. & Khalil, B. Using discrete wavelet transforms to analyze trends in streamflow and precipitation in quebec and ontario (1954–2008). *J. Hydrol.* **475**, 204–228 (2012).

[46] Cohen, J. *Stat. Power Anal. Behav. Sci.* 2nd edn. (Lawrence Erlbaum Associates, Hillsdale, NJ, 1988).

[47] Guyon, I. & Elisseeff, A. An introduction to variable and feature selection. *J. Mach. Learn. Res.* **3**, 1157–1182 (2003).

[48] Hyndman, R. J. & Koehler, A. B. Another look at measures of forecast accuracy. *Int. J. Forecast.* **22**(4), 679–688 (2006).

[49] Greer, M. Directional accuracy tests of long-term interest rate forecasts. *Int. J. Forecast.* **19**(2), 291–298 (2003).

[50] Blaskowitz, O. & Herwartz, H. Testing the value of directional forecasts in the presence of serial correlation. *Int. J. Forecast.* **30**(1), 30–42 (2014).

[51] Chawishborwornworng, C. et al. Bootstrap approach for quantifying the uncertainty in modeling of the water quality index using principal component analysis and artificial intelligence. *J. Saudi Soc. Agric. Sci.* **23**(1), 17–33 (2024).

[52] Sutanto, S. J. et al. Skill of large-scale seasonal drought impact forecasts. *Nat. Hazards Earth Syst. Sci.* **20**(6), 1595–1608 (2020).

[53] Ergüven, R., Aydin, A. B. & Avci, D. Drought forecasting using standard precipitation index and artificial intelligence models in the mediterranean region of türkiye. *Appl. Sci.* **15**(22), 12172 (2025).

[54] Sibiya, S. et al. Meteorological drought trend analysis and forecasting using a hybrid sg-ceemdan-arima-lstm model based on spi from rain gauge data. *Nat. Hazards Earth Syst. Sci.* **26**(1), 315–342 (2026).

[55] Pande, C. B. et al. A novel machine learning models for meteorological drought forecasting in the semi-arid climate region. *Appl. Water Sci.* **15**(6), 121 (2025).

